# The CaT stretcher: An open-source system for delivering uniaxial strain to cells and tissues (CaT)

**DOI:** 10.3389/fbioe.2022.959335

**Published:** 2022-10-18

**Authors:** Yushi Wang, Ryan Singer, Xinyue Liu, Seth J. Inman, Quynh Cao, Quan Zhou, Alex Noble, Laura Li, Aidee Verónica Arizpe Tafoya, Mouhanad Babi, Kjetil Ask, Martin R. Kolb, Scott Ramsay, Fei Geng, Boyang Zhang, Yaron Shargall, Jose Manuel Moran-Mirabal, Mohammadhossein Dabaghi, Jeremy A. Hirota

**Affiliations:** ^1^ Department of Medicine, Firestone Institute for Respiratory Health—Division of Respirology, McMaster University, Hamilton, ON, Canada; ^2^ School of Biomedical Engineering, McMaster University, Hamilton, ON, Canada; ^3^ Department of Materials Science and Engineering, University of Toronto, Toronto, ON, Canada; ^4^ Department of Chemistry and Chemical Biology, McMaster University, Hamilton, ON, Canada; ^5^ Centre for Advanced Light Microscopy, McMaster University, Hamilton, ON, Canada; ^6^ McMaster Immunology Research Centre, McMaster University, Michael G. DeGroote Centre for Learning and Discovery, Hamilton, ON, Canada; ^7^ W Booth School of Engineering Practice and Technology, McMaster University, Hamilton, ON, Canada; ^8^ Department of Chemical Engineering, McMaster University, Hamilton, ON, Canada; ^9^ Division of Thoracic Surgery, Department of Surgery, McMaster University, Hamilton, ON, Canada; ^10^ Department of Medicine, Division of Respiratory Medicine, University of British Columbia, Vancouver, BC, Canada; ^11^ Department of Biology, University of Waterloo, Waterloo, ON, Canada

**Keywords:** mechanotranduction, fibroblast, lung, epithelial cell, extracellular matrix

## Abstract

Integration of mechanical cues in conventional 2D or 3D cell culture platforms is an important consideration for *in vivo* and *ex vivo* models of lung health and disease. Available commercial and published custom-made devices are frequently limited in breadth of applications, scalability, and customization. Herein we present a technical report on an open-source, cell and tissue (CaT) stretcher, with modularity for different *in vitro* and *ex vivo* systems, that includes the following features: 1) Programmability for modeling different breathing patterns, 2) scalability to support low to high-throughput experimentation, and 3) modularity for submerged cell culture, organ-on-chips, hydrogels, and live tissues. The strategy for connecting the experimental cell or tissue samples to the stretching device were designed to ensure that traditional biomedical outcome measurements including, but not limited to microscopy, soluble mediator measurement, and gene and protein expression remained possible. Lastly, to increase the uptake of the device within the community, the system was built with economically feasible and available components. To accommodate diverse *in vitro* and *ex vivo* model systems we developed a variety of chips made of compliant polydimethylsiloxane (PDMS) and optimized coating strategies to increase cell adherence and viability during stretch. The CaT stretcher was validated for studying mechanotransduction pathways in lung cells and tissues, with an increase in alpha smooth muscle actin protein following stretch for 24 h observed in independent submerged monolayer, 3D hydrogel, and live lung tissue experiments. We anticipate that the open-source CaT stretcher design will increase accessibility to studies of the dynamic lung microenvironment through direct implementation by other research groups or custom iterations on our designs.

## 1 Introduction

The cell and tissue microenvironment plays a dominant role in the development, structure, and function of all living organisms by contributing biochemical, topographical, and mechanical cues to cells in 3D under static and dynamic conditions (McGowan, 1992; Suki et al., 2005; Roan and Waters, 2011; Brusatin et al., 2018; Xu et al., 2021). The human lung is an obvious example of a dynamic environment, facilitating ventilation for gas exchange with every inhalation and exhalation cycle performed throughout our lifetimes and under constant strain from transpulmonary pressure gradients. The 3D microenvironment transmits cues to lung cells under resting and breathing-induced strain that contributes to lung development, cell differentiation, surfactant release, smooth muscle function, immune responses, and repair processes (Wirtz and Dobbs, 1990; Sanchez-Esteban et al., 2001; Shkumatov et al., 2015; Gilpin et al., 2017; Zhou et al., 2018; Bai et al., 2022). The composition of the 3D microenvironment is perturbed during lung pathologies, aging, and infections and when combined with dynamic forces, can contribute to amplification of processes observed under static conditions (Booth et al., 2012; Wagner et al., 2014; Froese et al., 2016; Gilpin and Wagner, 2018; Shimbori et al., 2019; Bai et al., 2022). These studies highlight the importance of integrating both the 3D microenvironment and dynamic forces into *in vitro* and *ex vivo* lung cell biology studies in attempt to faithfully recapitulate the *in situ* lung microenvironment.

The 3D microenvironment impacts lung cell and tissue biology through biochemical, topographical, and mechanical cues. Biochemical cues can be soluble mediators including extracellular nucleotides and their metabolic products (Murata et al., 2014; Takahara et al., 2014; Tan et al., 2020) or be communicated through cell-cell and cell-ECM contacts. A dominant class of cellular receptors responsible for communicating between the extracellular and intracellular compartments are integrins, dimers consisting of diverse *α* and *β* subunits, that reside on cell surfaces to engage with ECM (Hynes, 2002; Bachmann et al., 2019). Integrins are organized into 24 different heterodimers based on known *α* and *β* subunits and further categorized based on structure, function, ligands, and cell and tissue expression patterns (Hynes, 2002; Bachmann et al., 2019). Within plasma membrane restricted focal adhesions, integrins connect on the extracellular side with motifs featured in ECM proteins and on the intracellular side with protein complexes that coordinate interaction with cytoskeleton proteins actin and myosin (Hynes, 2002; Totaro et al., 2018; Bachmann et al., 2019). Signaling downstream of integrins may activate mechanosensitive transcription factors and complexes including Yes-Associated Protein (YAP), Transcriptional co-activator with PDZ-binding motif (TAZ), and Linker of Nucleoskeleton and Cytoskeleton (LINC) (Totaro et al., 2018). YAP/TAZ signaling is a dominant mechanism for mechanotransduction and is able to upregulate genes that enable diverse cellular responses to changes in the microenvironment that may include cell proliferation, stretching, re-organization, and differentiation (Totaro et al., 2018). YAP/TAZ signaling in lung cells has been confirmed, with clear implications for fibroblast differentiation to myofibroblasts being impacted by the cell culture surface stiffness and composition (Wipff et al., 2007; Parker et al., 2014; Liu et al., 2015; Piersma et al., 2015). Explorations into YAP/TAZ signaling in lung cells has been facilitated by the use of hydrogel, a broad type of materials that possess a crosslinked polymer network and that swell when exposed to water. Hydrogels can be composed of ECM proteins from plant, non-human animal, or human organs and are able to provide biochemical, topographical, and mechanical cues to cells that would exist *in situ* ^27,28^ *.* Hydrogel cues can be tuned and have been exploited in lung cell biology in the context of mechanosensing (Marinković et al., 2013; Liu et al., 2015). Human lung derived ECM can be used to create hydrogels that share properties with naïve intact human lung and enable culture of lung cells in an accurate approximation of the *in situ* microenvironment (Smithmyer et al., 2019; De Hilster et al., 2020; Dabaghi et al., 2021a). Interestingly, cells experiencing particular microenvironments are able to “remember” their environment in a form of cellular memory that is likely important in healthy lung development and disease processes (Balestrini et al., 2012; Yang et al., 2014).

To model the dynamic forces that accompany breathing, commercial and custom cell and tissue devices have been developed (Al-Maslamani et al., 2021). Commercially available platforms are turn-key solutions that provide the ability to introduce stretch through either pressure or motor driven mechanisms, enabling dynamic forces to be applied to the 3D microenvironment. Custom devices designed by academic research groups have provided additional flexibility in experimental designs through open source hardware and software designs (Jain et al., 2010; Seriani et al., 2016; Mayer et al., 2019; Al-Maslamani et al., 2021; Cei et al., 2021; Correia Carreira et al., 2021; Daulagala et al., 2021; Doryab et al., 2021). The available devices vary in their method of introducing stretch, with uniaxial, biaxial, multiaxial, or radial, and are motor or pneumatic driven (Kurata et al., 2019; Shiwarski et al., 2020; Kah et al., 2021). Multiaxial stretch occurs at the alveolar unit and biaxial or uniaxial stretch may not completely recapitulate the *in situ* environment (Nossa et al., 2021). Custom designs are increasingly more approachable due to the accessibility of rapid prototyping technologies such as 3D printing, economical computing and electronics such as Raspberry Pi and Arduino devices, and open-source repositories for hardware and software design plans. An excellent review of *in vitro* lung devices that introduce mechanical forces and exposures has recently been published that provides comparisons of the different technologies available (Nossa et al., 2021).

From this perspective, we set out to develop the Cell and Tissue (CaT) stretcher, as an open-source device amenable to studying lung cell and tissue microenvironments under static and dynamic stretch conditions. The CaT stretcher was built off a foundation of open source design with constraints that included the need for 1) a dynamic system that could model diverse breathing patterns, 2) scalable to enable efficient experimentation with simultaneous control and treatment groups possible, 3) modular design to allow for different cells and tissue formats to be studied, and 4) suitable for 2D and 3D experimentation. We sought to develop a system that could incorporate diverse sample types including traditional submerged monolayer cultures, air-liquid interface cultures, organs-on-chips, 3D hydrogels, and live explanted tissue. The strategies for connecting the experimental cell or tissue samples to the CaT stretcher were designed to ensure that traditional biomedical outcome measurements including, but not limited to microscopy, soluble mediator measurement, and gene and protein expression remained possible. Lastly, to increase the uptake of the device within the community and future adaptability, the system needed to be open-source and built with economically feasible and available components. The net result of these constraints is the present technical report on the programmable CaT stretcher and a suite of cell and tissue chip designs that provides both scalability and modularity. We anticipate that the CaT stretcher design will increase accessibility to studies of the dynamic lung microenvironment through direct implementation by other research groups or custom iterations on our open source design.

## 2 Materials and methods

### 2.1 Human ethics

Procurement of primary human fibroblasts and lung tissue was approved by Hamilton integrated Research Ethics Board (HiREB 5099T, 5305T) and from consented subjects.

### 2.2 System design

*A priori* design objectives were defined to constrain system development and ensure broad applicability in the biomedical research laboratory. The overarching goal was to develop and open-source, programmable, cell, and tissue stretching device that could be operated on a lab bench (ambient temperature and humidity), biosafety cabinet, or incubator (37°C and 100% humidity), with a limited requirement for user expertise in engineering and software development and standard power requirements. The desired mechanical strain introduced to cells and tissues was required to be consistent with *in situ* forces experienced in living lung in a variety of healthy and disease states. Modularity was considered important for different applications spanning from submerged monolayer culture, air-liquid interface culture, 2D/3D hydrogel cultures, and live tissue strips. Scalability was also prioritized for the ability to perform multiple replicates or experimental conditions at the same time. The result was a programmable mechanical device with oscillations that could model 4–60 breaths per minute with a strain of 4%–40%, suitable for use in diverse lab environments, and amenable to multiple *in vitro* and *ex vivo* experimental designs, with scalability for performing at least 8 simultaneous experimental replicates (Table 1 CaT Stretcher Specifications). Consistent with open-source practice, a complete list and location of design files for the electronics, mechanical device, and sample mounts is provided (Table 2 CaT stretcher accessory design and software files) and accompanied by a Bill of Materials with vendors and cost estimates in CAD (Table 3 CaT stretcher bill of materials) with a separate description of each major part (Table 4 Part Descriptions). A GitHub repository hosts all required documentation and assembly manuals (https://github.com/crystalliu314/CaT_Stretcher).

**TABLE 1:** CaT stretcher specifications.

Maximum Load	500 N
Effective Stroke	200 mm
(Chambers/Samples Length + Maximum Stretch Length)	
Optimal Operating Velocity Range	3–300 mm/min
Space for Mounting Chambers/Samples	Short 200 mm
	Long 400 mm
Stretch Patterns	Sinusoidal, Square, + Retention
Dimensions	400 mm (L) x 300 mm (W) x 80 mm (H)
Weight	3.5 kg
Operating Systems Available	Windows, Linux, MacOS

**TABLE 2:** CaT stretcher design and software files.

File name-description	File format	License type	Location of file
SampleMount-SubmergedMonolayer	STL′	CC-BY 4.0	GitHub Folder—https://github.com/crystalliu314/CaTStretcher/tree/main/Mechanical%20Design/Custom%20Parts%20Design%20Files
SampleMount-SubmergedMonolayerSchamberact	STL	CC-BY 4.0	
SampleMount-OrganOnChip	STL	CC-BY 4.0	
SampleMount-HydrogelPillar	STL	CC-BY 4.0	
SampleMount-TissueClamp	STL	CC-BY 4.0	
Firmware		CC-BY 4.0	GitHub—https://github.com/crystalliu314/CaTStretcher/tree/ma in/Code/CaTArduino
Software GUI		CC-BY 4.0	GitHub—https://github.com/crvstalliu314/CaTStretcher/tree/main/Code/Cell Stretcher UI Pi V2
Printed Circuit Board		CC-BY 4.0	GitHub—https://Aithub.com/crvstalliu314/CaTStretcher/tree/main/Electrical%20Design/PCB
Custom parts—3D Printed parts	STL	CC-BY 4.0	GitHub—https://github.com/crystalliu314/CaTStretcher/tree/main/Mechanical%20Design/Custom%20Parts%20Design%20Files/3D%20Printing
Custom parts—Casting	STEP	CC-BY 4.0	GitHub—https://github.com/crystalliu314/CaTStretcher/tree/main/Mechanical%20Design/Custom%20Parts%20Design%20Files/Casting
Custom parts—Machining	STEP	CC-BY 4.0	GitHub—https://github.com/crystalliu314/CaTStretcher/tree/main/Mechanical%20Design/Custom%20Parts%20Design%20Files/Machining

**TABLE 3:** CaT stretcher bill of materials.

Part number	Description	Quantity	Unit price (CAD)		Vendor	Price (CAD)	
Mechanical components							
100	Slider rail assembly	1	$	62.00	jian An Machinery	$	62.00
101	End attachment bracket	1	$	5.00	Custom Machined	$	5.00
102	Rail attachment bracket	1	$	1.00	3D Printed	$	1.00
103	Aluminum extrusion 2020 x 450 mm	2	$	15.00	Amazon/AliExpress	$	30.00
104	PDMS Wells mounting plate	4	$	1.00	3D Printed	$	4.00
200	PDMS	Wells	8	N/A	Custom casting		
301	Well mounting pins - short	50	$	0.52	McMaster Carr	$	26.00
302	Well pins - long	N/A		McMaster Carr			
303	M4X12	4	$	0.10	McMaster Carr	$	0.40
304	M5X10	28	$	0.20	McMaster Carr	$	5.60
305	M5X16	4	$	0.08	McMaster Carr	$	0.32
311	M4X30	4	$	0.27	McMaster Carr	$	1.08
312	M4 nuts	4	$	0.02	McMaster Carr	$	0.08
Electronics							
400	Electronics control box assembly	1		N/A			
401	Arduino Uno	1	$	5.49	Amazon/AliExpress	$	5.49
402	PC8	1	$	15.00	JLCPC8 + Custom Wort	$	15.00
403	Raspberry Pi 4	1	$	75.00	CanaKit	75.00	
404	Raspberry Pi 7″ Screen	1	$	54.00	Amazon/AliExpress	$	54.00
405	NEMA23 Stepper	1	$	10.00	Amazon/AliExpress	$	10.00
406	Emergency Stop	1	$	2.00	AliExpress	$	2.00
407	Electronics housing base	1	$	2.00	3D Printed	$	2.00
408	Electronics housing front cover	1	$	1.00	3D Printed	$	1.00
409	Electronics housing back cover	1	$	1.00	3D Printed	$	1.00
410	Electronics housing button cover	1	$	0.50	3D Printed	$	0.50
411	ElOmm cooling fan	1	$	2.30	Amazon/AliExpress	$	2.30
306	M3 nuts	7	$	0.05	McMaster Carr	$	0.35
307	M3X14	7	$	0.05	McMaster Carr	$	0.35
308	M2.5 8 mm female-female standoffs	4	$	2.05	McMaster Carr	$	8.20
309	M3X5	9	$	0.07	McMaster Carr	$	0.63
310	M2.5X5	4	$	0.28	McMaster Carr	$	1.12
Miscellaneous							
901	12 V power supply with USB	1	$	12.87	Amazon/AliExpress	$	12.87
902	Keyboard + Mouse	1	$	40.00	Amazon/AliExpress	$	40.00
903	USB C Pi Power supply	1	$	10.00	Amazon/AliExpress	10.00	
904	USB A to B cable	1	$	0.90	Amazon/AliExpress	0.90	
905	USB B female to male right angle adaptor		1	$	2.00	Amazon/AliExpress	2.00
906	MicroHDMI to HDMI cable	1	$	3.00	Amazon/AliExpress	3.00	
907	Micro SD card	1	$	6.50	Amazon/AliExpress	6.50	
908	Male to female standard HDMI angled adaptor	1	$	2.00	Amazon/AliExpress	$	2.00
909	Male to female barrel connector (5.5 mm OD, 2.1 mm ID)	1	$	5.00	Amazon/AliExpress	$	5.00
910	N42 3/8″ diameter 3/32″ thick neodymium magnets	16	$	0.55	Simple Signman	$	8.80
911	N42 3/8″ diameter 1/16″ thickneodymium magnets	16	$	0.55	Simple Signman	$	8.80
912	Single edge razor blades	10	$	0.12	Uline	$	1.20
Total							$ 415.49

**TABLE 4:** Part descriptions.

Part Number	Part	Description
Mechanical components		
100	Slider rail assembly	Ball screw slider rail platform, the base of the entire stretcher, ball screw converts motor rotary motion to linear motion
101	End attachment bracket	Custom machined out of aluminum, attach one side of aluminum extrusion rail onto the end of the slider rail assembly
102	Rail attachment bracket	3D printed out of PLA, attach the moving side of aluminum extrusion rail onto the moving platform on the slider rail assembly
103	Aluminum extrusion 2020 x 400 mm	Off-the-shelf aluminum extrusion, 2020 profile (20 mm × 20 mm), a base for attaching cell and tissue chambers onto the moving platform
104	PDMS Wells mounting plate	3D printed out of PLA, attach the PDMS wells to the aluminum extrusions using well mounting pins, customizable size and shape
200	PDMS Wells	Casted out of PDMS, housing live cell and tissue to be stretched
Electronics		
400	Electronics control box assembly	Integrated control box, including the microcontroller, custom PCB, raspberry pi acting as a computer, and a 7″ screen for user operation
401	Arduino Uno	Microcont roller board
402	PCB	Integrated electronics for power regulation, motor control, and physical switch
403	Raspberry Pi 4	Linux computer for user interface with Ardui no Uno
404	Raspberry Pi 7″ Screen	Display for user interface
405	NEMA23 Stepper	Motor that provides motion to the strecher
406	Emergency Stop	Safety feature for stopping machine motion immediately

**FIGURE 1:**
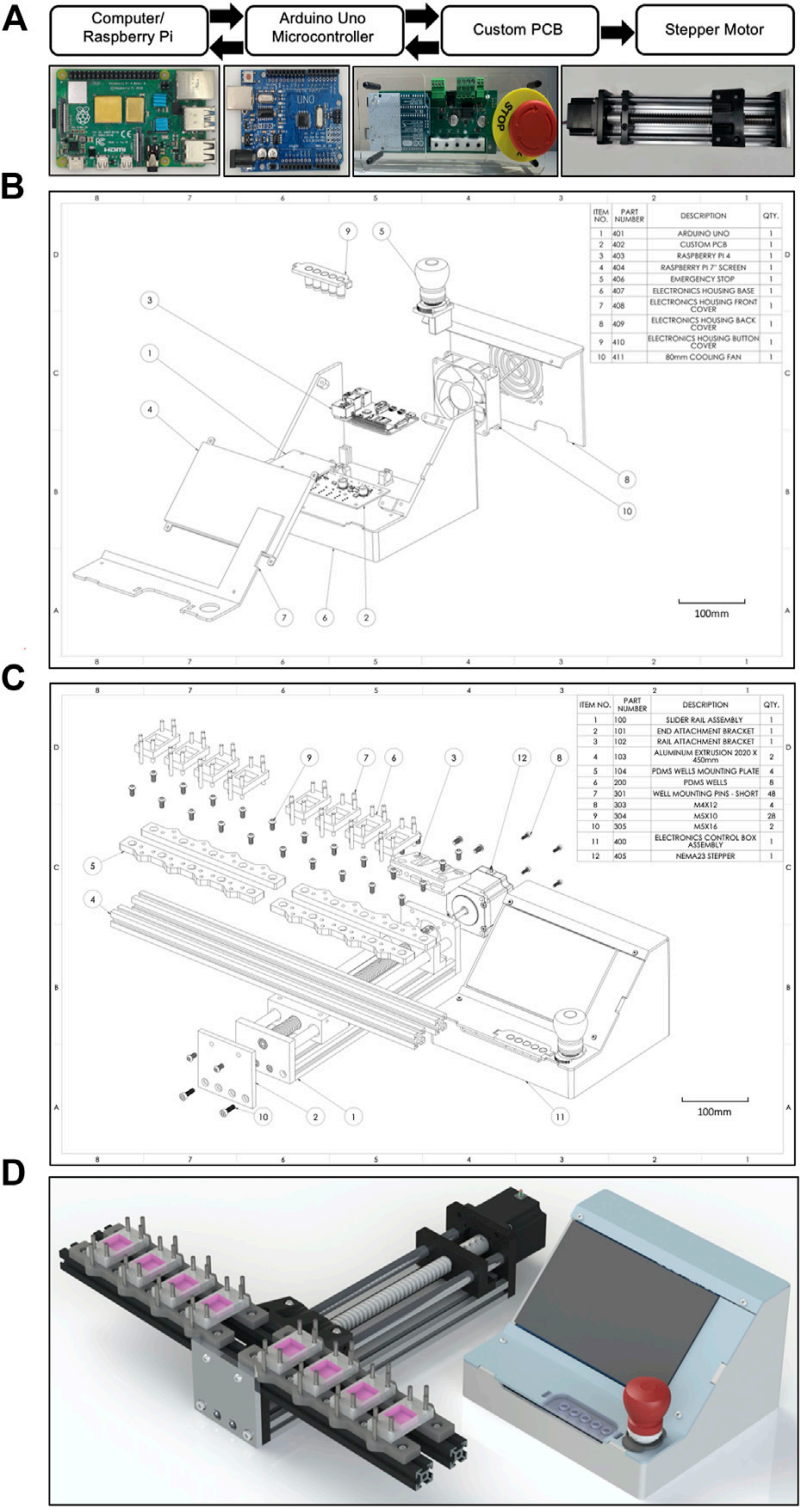
Design overview and schematics of the CaT stretcher system. **(A)** Electronic and mechanical motor integration and paths of communications. Text bubbles are presented above the main CaT stretcher components with corresponding photographs below. **(B)** Explosion diagram of the CaT stretcher controller with numbered parts listed**. (C)** Explosion diagram of the CaT stretcher mechanical device with numbered parts listed. The presented configuration is with 400 mm rails, 40 mm well mounting pins, and 8 individual 4 cm^2^ submerged monolayer chip designs. **(D)** 3D CAD rendering of the CaT stretcher device, configured as described above.

The system is a configuration that includes a Raspberry Pi 4 computer connected to an Arduino Uno microcontroller that is itself connected to a custom printed circuit board (PCB) to control a NEMA 23 stepper motor (Figure 1A). The PCB houses 5 external user buttons (defined below in “Electronics”) that control a Trimeric TMC2209 silent stepper motor driver on the PCB. The mechanical portion of the CaT stretcher consists of a ball screw driven moving platform which slides along a pair of guide shafts. Mechanical actuation is provided by a stepper motor, producing linear uniaxial motion along the guide shafts. The Arduino Uno is interfaced with a computer running the custom CaT stretcher software application *via* USB, which can be run on Windows, Mac OS, or Linux. A benchtop version of the machine uses a Raspberry Pi 4 with a Raspberry Pi 7-inch display. Labeled explosion diagrams of the electronic controller (Figure 1B) and mechanical stretcher (Figure 1C) provide listed parts and quantities. Custom 3D finite deposition modeling was used to fabricate the electronic controller housing and rail bracket mounting plates and machining for the slider rail assembly. A rendering of a final assembled device is provided in a configuration for 8-plex submerged monolayer culture experimentation (Figure 1D). All software, design, and assembly guide files are open-source and links to the GitHub repository are provided in Table 2. Individual design components (mechanical device, electronics, controller software, and cell and tissue chips) are described in greater detail below.

### 2.3 Electronics

The CaT stretcher is centered around an Arduino Uno microcontroller board, which is the main controller for the machine and manages communications with the Raspberry Pi 4 (Figures 1A,B). The electronics of the CaT are integrated in one PCB which inserts into the Arduino Uno’s female header pins as a shield. The system accepts 12 V DC power input from a generic wall adapter. A 5 V power rail for control electronics is derived using a TPS56200 buck converter. It is connected to the PCB through female sockets on the side, allowing the Arduino board to be swapped without additional tools. A TMC2209 stepper driver is used to control the stepper motor, providing up to 2A root mean square drive current. Five physical buttons are included on the board for the users to operate the machine, named JOG FORWARD, JOG BACK, TARE, START, and AUX (auxiliary, programmed in software to bring the machine out of the STOP state). Three LED indicators are also included to provide an indication of machine states, named STOP, RUNNING, and AUX. The CaT stretcher includes an emergency stop button which plugs into the PCB as a switch to provide a safety feature that when pressed, stops the motor immediately and de-energizes the unit. The schematics of the PCB as manufactured can be found in the GitHub repository dedicated to this manuscript (Table 2).

### 2.4 Controller software

**FIGURE 2:**
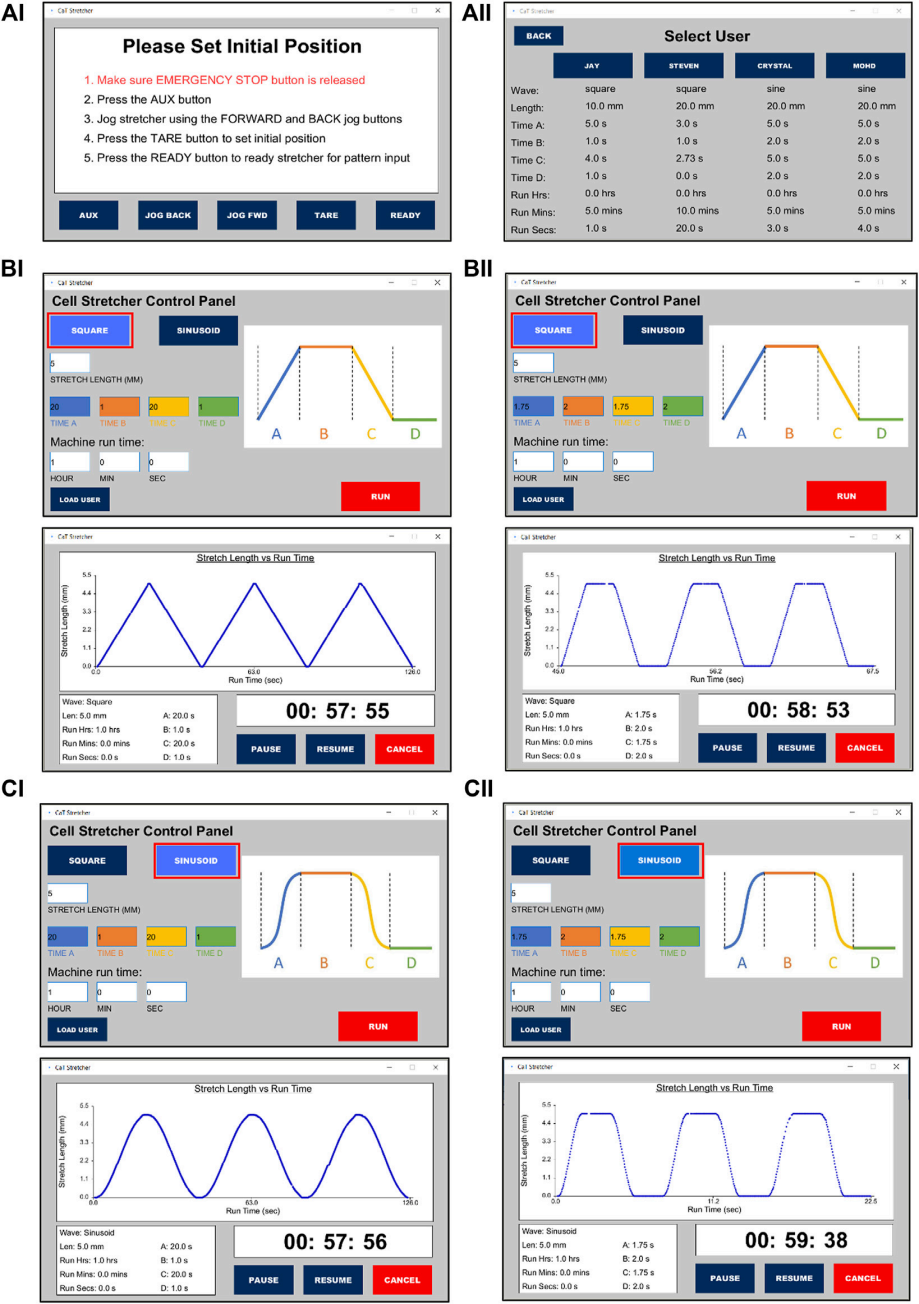
CaT stretcher controller software user interface for programming and loading saved user experimental protocols. **(Ai)** CaT software home screen. **(Aii)** User profiles and saved experimental protocols. Programming a **(B)** square and **(C)** sinusoid wave form stretch of 5 mm under distinct time conditions (top panels) with the real-time recordings and shape of the waveforms presented during stretch (bottom panels). **(Bi,Ci)** Ramp = 20 s, Pause = 1 s, Return ramp = 20 s, Pause = 1 s. **(Bii,Cii)** Ramp = 1.75 s, Pause = 2 s, Return ramp = 1.75 s, Pause = 2 s.

User control over the CaT stretcher is done through a graphical user interface (GUI), which operates through three different primary states: initialization/setup, parameter setting, and running (Figure 2; Supplementary Video S1). Upon startup, the user is presented with a dropdown window listing all available serial/USB ports. The port connected to the Arduino must be selected to operate the machine *via* software.

Once the correct port has been selected, the setup screen will appear. Here, the user can adjust the machine to the desired starting position following provided on-screen instructions using either the software or physical buttons. Pressing the READY button will transition the GUI to the parameter setting state where the user can adjust multiple stretch settings such as wave geometry, stretch length, motion, and dwell durations, as well as total stretch time. Custom settings can be saved in four built-in user profiles which can be accessed *via* the LOAD USER button on the setup screen. To access or change a saved profile, the user of interest is selected. From the saved setting location, a stretch can be started directly with the pre-saved values. Pressing RUN from the parameter setting state will start the stretch with the selected specifications. Once the stretch session ends or is terminated by the user, the machine will jog back to the initial set position to allow for specimen removal. If the emergency stop button is pressed at any point during the operation, the machine will enter the stop state. The user can exit this state and resume where they left off by following the on-screen instructions.

The software package for the CaT stretcher includes two components: The Arduino controller firmware (written in Arduino IDE using C++) and the GUI application (written in Processing IDE using Java). The Arduino Uno and firmware is the central hub responsible for direct communication with the hardware components and the GUI. The Processing GUI serves to provide a front-end interface that allows non-technical users to operate the machine. Once the user starts stretching, the GUI software will loop quickly and calculate instantaneous speed and next position values on each loop. These values will then be sent to the Arduino firmware which translates them to number and frequency of steps.

### 2.5 Mechanical stretcher

**FIGURE 3:**
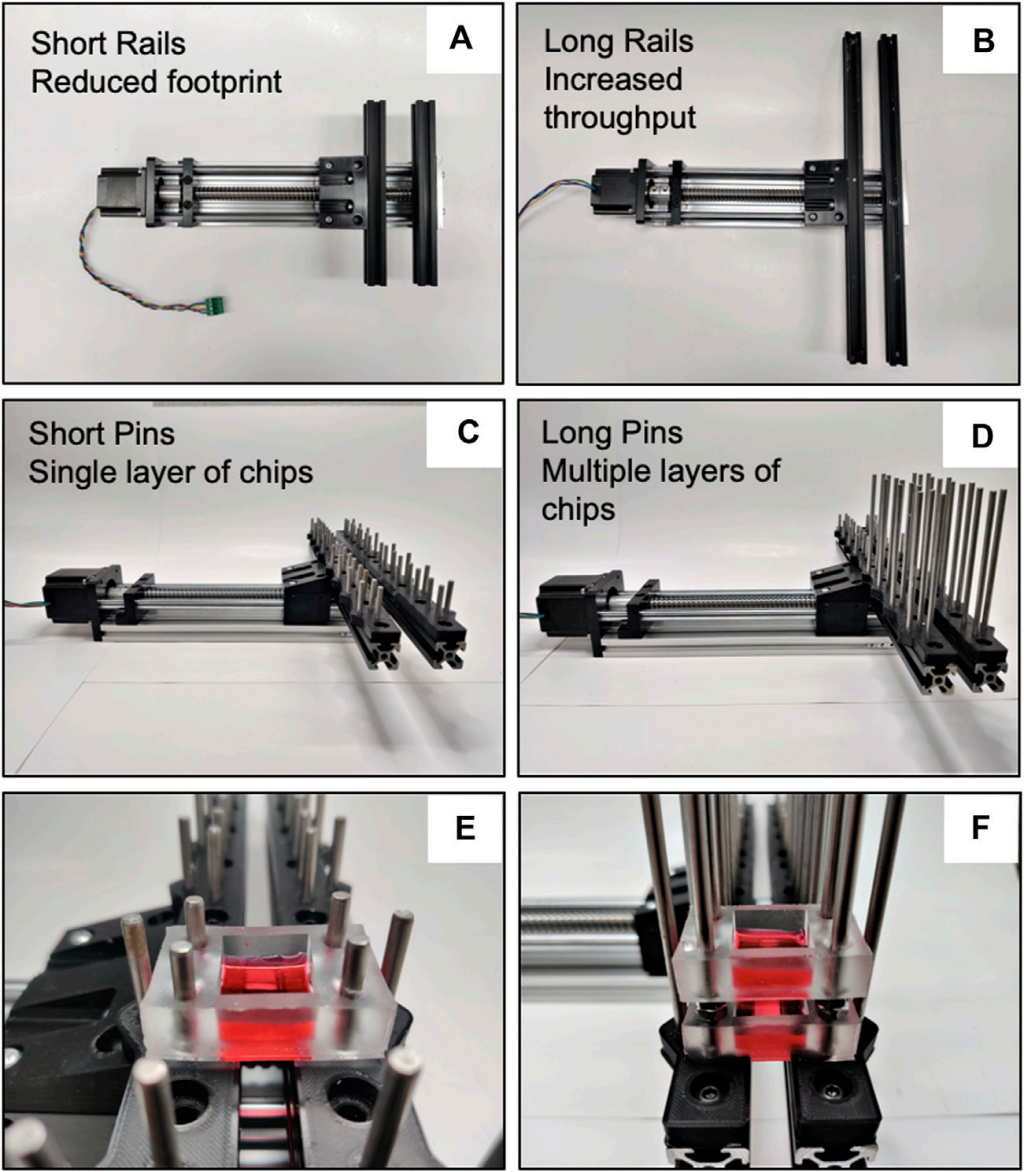
CaT stretcher rail system modularity and scalability*.* **(A)** Presented with 200 mm rails for a reduced footprint. **(B)** Presented with 400 mm rails for increased throughput. **(C)** Short (50 mm pins) for a single layer of chips. **(D)** Long (100 mm) pins for multiple layers of chips for scalability in experimental trials. **(E)** Demonstration of chip attachment to short pins (4 cm^2^ submerged monolayer chip shown). **(F)** Chip stacking with long pins. All rail and pin configurations are compatible with all chip designs presented in the manuscript.

The mechanical components of the CaT stretcher are designed around a ball screw driven moving platform (Figure 1C). The rail has an effective stroke that allows for stretch of up to 200 mm. The ball screw is 16 mm OD, 5 mm lead. The use of an integrated linear slide rather than individual rails and lead screw components simplifies the assembly steps. The platform is driven by a NEMA 23 stepper motor, which has a torque rating of 0.65 Nm. This motor was deemed sufficient for our applications based on estimates of returning forces that our chip designs exert on the motor under maximal strain scenarios, which requires around 0.35 Nm torque. The stepper motor is fixed to the linear slide using M4 screws and coupled to the ball screw to produce linear motion. Aluminum extrusions with T-slots are used for mounting sample fixation attachments through 3D printed attachment plates (Figure 1C). Any mounting on or from the aluminum extrusions is done using M5 screws threaded through T-nuts seated within the extrusions. As different test samples may require different size attachments, this design allows maximum flexibility in the type of samples and attachment methods the machine could accommodate. To accommodate different environmental constraints and throughput requirements the selected aluminum extrusions can be increased in length (Figure 3). Shorter rails reduce the footprint to accommodate smaller incubators and constrained biosafety cabinet space (Figure 3A). Longer rails increase the number of cell and tissue chips that can be attached for a given experiment to increase throughput (Figure 3B). The cell and tissue chips are attached with stainless steel mounting pins (short—50 mm) that have also been chosen to be flexible for footprint and throughput. Longer pins (long—100 mm) enable opportunities to scale the experimentation by stacking cell and tissue chips vertically (Figures 3C–F). The entire system can be sprayed with 70% ethanol for sterilization prior to use as needed.

### 2.6 Cell and tissue chip fabrication

**FIGURE 4:**
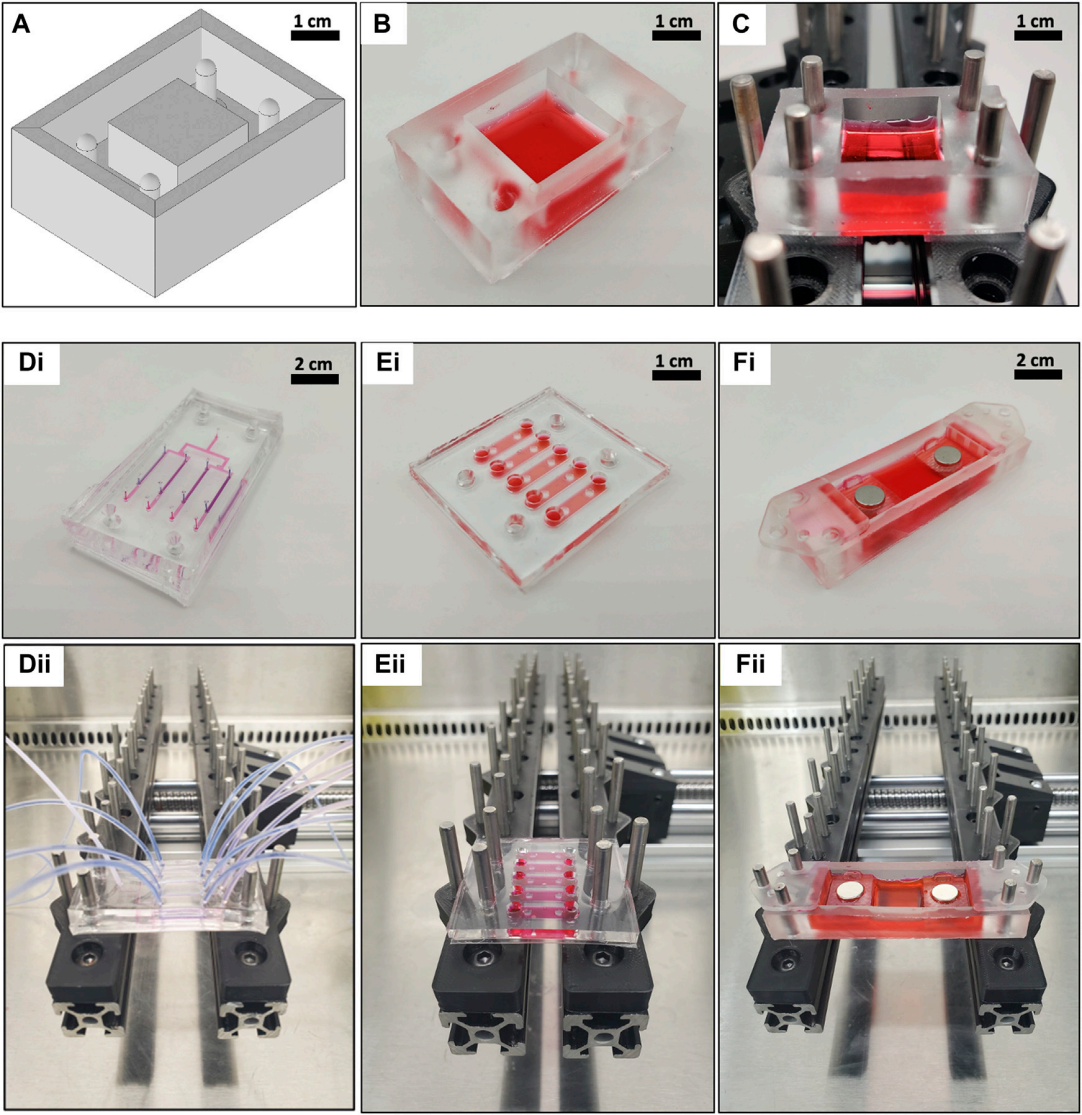
CaT stretcher chips provide modularity for diverse cell and tissue experimental designs. **(A–C)** Sample workflow for 3D CAD chip design and fabrication. For more details on fabrication on all chips, see Table 2 for design files and Supplementary Figure S2–S6 for visualized fabrication steps. **(A)** A negative mold is designed in CAD software and printed with a 3D resin printer. **(B)** PDMS is cast into the mold and removed to create the desired chip (filled with red DMEM for display purposes). **(C)** Chip attachment to the rail and pin system of the CaT stretcher. **(D–F)** Alternate chip designs compatible with the CaT stretcher rail and pin system. **(D)** Perfusable organ-on-chip with apical and basal compartments separated by a membrane amenable to cell culture. Blue fluid represents an apical compartment, pink fluid represents a basal compartment. Liquid-liquid cultures and air-liquid cultures are possible. **(E)** 3D hydrogel pillar chip. **(F)** Tissue clamp chip with magnets. Figure panels (i) represent built configurations on bench and (ii) represent configurations attached to the CaT stretcher rail and pin system.

We present a variety of chips to showcase the basic capabilities of CaT Stretcher attachments (Figure 4). All chips were fabricated *via* soft lithography using 3D printed negative molds (Form 3B—Formlabs) or photolithography-based master molds. Conventional photolithography was used to fabricated master molds where greater resolution was of interest. Molds were filled with polydimethylsiloxane (PDMS, Sylgard 184, Dow Corning, Midland, and MI) prepared at a 20:1 (wt/wt) base-to-curing agent ratio to provide optimal stiffness and compliance for attachment and stretch, respectively, on the CaT stretcher. We measured the Young’s Modulus of the 1:20 and 1:10 ratios of base-to-curing agent, observing values of 0.52 MPa (0.07-SD) and 5.6 MPa (0.69—SD), respectively. PDMS provides an optically clear, biocompatible material, with tunable mechanical properties that can be stretched and coated for cell culture applications. Each chip is fabricated from PDMS either as one piece or multiple pieces to be bonded into one. Chip designs contained holes for attachment to the CaT stretcher *via* stainless steel pins (Figure 3; Supplementary Videos S2, S3).

The preparation process was similar for all chip designs with negative molds of the desired geometry of choice produced first. 3D printed molds were designed using Autodesk Inventor software and exported as stereolithography (.STL) files. These files were sent to a Form 3B Formlabs stereolithography 3D printer to fabricate the desired part with system settings aided from the Formlabs build software (see Table 2 for all design related files). Regarding photolithography-based master molds, the designs were prepared in Autodesk Inventor software and film photo masks were used to fabricate master molds. To facilitate the release of PDMS replicated from photolithography-based master molds, Chemical vapor deposition technique was used to coat a thin and uniform layer of a trichloro (1H,1H,2H,2H-perfluorooctyl) silane (Sigma Aldrich, Product number: 448931-10G) on each master mold (Dabaghi et al., 2019). Before depositing the silane compound, each master mold was exposed to oxygen plasma (500 mTorr, Harrick Plasma cleaner, 15 W) for 90 s to activate the surface of the master mold (Dabaghi et al., 2019).

Following mold fabrication, PDMS was mixed at the 20:1 ratio followed by placement in a vacuum chamber at a pressure of ∼50 mm Hg for 30 min to remove any bubbles before being poured into molds. Each PDMS casting was incubated at 70°C in a level-surface oven for 6 h. To remove chips from molds, they were gently freed from the mold edges using a scalpel before being peeled off completely with care taken to not tear the PDMS. In some instances, flame activation of the PDMS surface was performed to increase bonding strength (Ghaemi et al., 2018). Flame activation required a butane torch to be brought near (∼1.5 cm for a standard butane hand torch) a PDMS surface with the hot point of the torch flame in contact with the surface for 10 s. Fabrication steps and visual workflows for all chip designs are provided in Supplementary Figures S2–S6.

### 2.7 Coating strategies to improve cell adhesion and proliferation

To prepare the surface of PDMS for cell adhesion, dopamine hydrochloride (Sigma, ON, Canada, Product number: H8502) with a concentration of 1 mg/ml was dissolved in phosphate-buffered saline (PBS) and the pH was adjusted to 8.5 using 1 M Sodium Hydroxide (NaOH). Then, submerged monolayer chips were incubated with this solution at room temperature for 24 h as described in our previous work to create a poly-dopamine (PDA) surface (Dabaghi et al., 2021b). After PDA coating, all devices were thoroughly rinsed with PBS (pH = 7.2–7.4) to remove any unattached PDA. PDA coating was followed by incubation with a solution of 25 µg/ml fibronectin bovine plasma (Sigma, ON, Canada, Product number: F4759-1 MG) and 50 µg/ml type I rat tail collagen (Advanced BioMatrix, CA, United States, product number: 5056-20 ML) together at 4°C for 24 h. Gelatin coating was performed *via* incubation with 1 mg/ml type A gelatin from porcine skin (Sigma, ON, Canada, Product number: 924504) at 60°C for 24 h. Collagen/fibronectin coating and gelatin coating were performed similarly in the absence of PDA coating for comparison purposes. After coating, chips were rinsed with PBS to remove unbonded molecules.

### 2.8 Cell culture and staining

Primary human lung fibroblast cells (HLFCs) were directly isolated from human lung tissues and expanded in DMEM cell culture media (ThermoFisher, Canada, product number: 11965-118) with 10% fetal bovine serum (FBS) (Wisent Inc., Saint-Jean-Baptiste, Quebec, Canada, product number: 080-450) and 1% penicillin-streptomycin (Gibco, United States) and used within the first seven passages of cell culture. Prior to seeding the HFLCs in all PDMS chips for experiments, the chips were exposed to UV in a biosafety cabinet for 30 min. PDMS chips used in hydrogel experiments were autoclaved before adding hydrogels. For both monolayer and 3D hydrogel experiments, a LIVE/DEAD™ Cell Imaging Kit (Catalog number: R37601, ThermoFisher, Mississauga, Ontario) was used to assess the cell viability.

#### 2.8.1 Monolayer experiments

For cell monolayer experiments, the HLFCs were seeded at a density of ∼1.5 ˣ 10^4^ cells/cm^2^ into either a single 2 cm^2^ chip (Supplementary Figure S2) or a multi-chamber chip (Supplementary Figure S3). Quantification of cell density was performed *via* fluorescent imaging by dividing live cell stain by the total growth surface area. An organ-on-chip design is also presented as an experimental design option (Supplementary Figure S4).

#### 2.8.2 Hydrogel experiments

HLFCs were added to a collagen pregel solution at a density of 1 × 10^6^ cells/ml. Neutralized bovine type I collagen solution (∼5 mg/ml) was purchased from Advanced Biomatrix (Cat# 5074, San Diego, CA, United States). First, the collagen solution was diluted by DMEM to the concentration of 3 mg/ml and HLFCs were added to a 4°C-collagen solution. After mixing the pregel solution and the cells, they were injected into the hydrogel pillar chips (Supplementary Figure S5) taking care to avoid introduction of air bubbles. Then, the chips were stored in an incubator for 1 h to allow for collagen gelation. Following gelation, the chips were submerged in prepared DMEM as described above and kept in an incubator until they were used for experiments.

#### 2.8.3 Staining

A NucBlue™ Live ReadyProbes™ Reagent (Hoechst 33342) from Invitrogen™ was used to permanently stain nuclei following manufacturer directions. Fixation was performed by first washing twice with PBS and then between each staining step. Fixing required 15 min incubation with 10% buffered neutral formalin. Fixed samples were permeabilized by 10 min incubation with 0.5% Triton X-100. A 1 h incubation with 3% bovine serum albumin (BSA) was used to reduce non-specific staining. Samples were incubated for 24 h at 4°C with primary antibodies in 1% BSA, followed by 24 h incubation at 4°C with secondary antibodies, DAPI, and phalloidin in 1% BSA. Fixed cells were stained with a monoclonal anti-α-smooth muscle (αSMA) actin antibody (Sigma Aldrich, catalog number: A2547). An ThermoFisher EVOS M7000 microscope with GFP, DAPI, and Texas Red cubes was used for widefield imaging of cells. A Nikon A1R HD25 inverted microscope was used for confocal imaging. To quantify the expression of αSMA, a region of interest with the same surface area between the left and right pillars of the hydrogel were selected for mean gray value quantification for each sample by ImageJ. All values were normalized to the mean αSMA expression of the static control samples.

### 2.9 Lung tissue preparation and stretching protocol

#### 2.9.1 Preparation

A two-piece slicing box (31 × 30 mm), razor blade holder, tissue clamps (tops and bottoms), and 60 × 20 mm well molds were 3D printed using the Form 3B resin printer. The entire procedure for lung slice generation and connection to the CaT stretcher is outlined visually in Supplementary Figure S7.

Two solutions of PDMS were separately prepared: a 10:1 mixture to cast around blades within the razor blade holder and a 25:1 mixture to fabricate a soft base for cutting. A 3 mm thick portion of the 25:1 mixture was poured into a petri dish to let cure at 70°C for 6 h. Once cured, a 30 × 30 mm square base piece was cut out. Next, a 4% weight to volume solution of low-temperature gelling agarose (Sigma, ON, Canada, product number: 39346-81-1) was prepared using HBSS (Thermo Fisher, Canada, product number: 14025092) as the solvent.

The metal clips covering the blunt end of nine single-edge razor blades were removed by gently inserting a flat laboratory spatula between the clip and the blade. Force was applied to pry away each clip while being careful of the sharp blade. All nine blades were inserted into the slots of the razor blade holder blunt-end first. With the holder supporting the blades in 3 mm spacing, a 5:1 mixture of PDMS was poured into the razor blade holder and around the blades while avoiding overflow. The PDMS was allowed to cure at 70°C for 6 h. The final product fabricated was nine evenly spaced razor blades held in place in the blade holder by surrounding PDMS.

Printed tissue clamps were then coupled with magnets using hot glue (product number: 8501502, Gorilla, Cincinnati, OH, United States). Neodymium magnets used were grade N42 and 3/8″ in diameter. Magnets of 3/32″ thickness (product number: D006P01, Simple Signman, Saint-Hyacinthe, QC, Canada) were used with clamp tops and those of 1/16” thickness (product number: D006P02, Simple Signman, Saint-Hyacinthe, QC, Canada) were used with clamp bottoms.

#### 2.9.2 Tissue cutting

The 30 × 30 mm PDMS square base piece was inserted into the inner slicing box, which was then placed into the outer slicing box. A strip of clear tape was wrapped under and along the sides of the inner slicing box protruding out of the box assembly as a makeshift handle for easier removal following cutting. The tissues were then cut roughly into a 30 × 30 × 10 mm geometry and placed into the inner slicing box on top of the PDMS base. The agarose solution was poured around the tissue and allowed to set in an ice box for approximately 15 min. Once the agarose had set, the inner slicing box was removed from the outer slicing box by lifting the clear tape and the razor blades were lined up with their appropriate slots. Using a gentil back and forth sawing motion, the blades were forced through the tissue. Cutting was continued until the blades sliced through the tissue and deeply into the PDMS base, which ensured that each slice was separated from the other. The 3 mm tissue slices were carefully removed with forceps from between the razor blades.

#### 2.9.3 Tissue clamping

With the CaT stretcher set up in the desired experimental setting (e.g., biosafety cabinet), PDMS wells were inserted onto the pins. Clamp bottoms were inserted onto the pegs and each well was filled with 3 ml of DMEM cell culture media (ThermoFisher, Canada, product number: 11965-118) with 10% fetal bovine serum (FBS) (Wisent Inc., Saint-Jean-Baptiste, Quebec, Canada, product number: 080-450) and 1% penicillin-streptomycin. Tissue slices were laid evenly between both clamps in each well. A clamp top was then inserted along the guide rails to fully hold one side of the slice. Using forceps, the tissue slices were gently pulled to their original length (defined by the cutting box) and installed under the magnetic clamps. This avoids any initial tissue sag that may affect the actual strain experienced by tissues during stretch. Lastly, 3 ml of additional DMEM solution was added to each well to fully submerge the tissues making sure to avoid any leaking or overflow.

#### 2.9.4 Post stretching

Following stretch protocol, tissues were gently released by removing the clamp tops using forceps. Each tissue slice was then carefully lifted out of the wells and the clamped portions were cut away using a single-edge razor blade to isolate the region of the tissue that had undergone strain and had not been clamped.

### 2.10 Statistical analysis

GraphPad Prism 9 (GraphPad Software Inc., United States) was used for graph generation and statistical analyses. The data was expressed in terms of mean and standard errors of the mean (SEM). One-way ANOVAs with Bonferroni correction for multiple comparisons test were used to compare the means of experimental groups. Where only two groups existed, unpaired t-tests were used to compare the means. Differences were considered statistically significant when *p* ≤ 0.05. * = *p* < 0.05, ** = *p* < 0.01, *** = *p* < 0.001, **** = *p* < 0.0001.

## 3 Results

### 3.1 The development of the open-source cell and tissue stretcher

To model the dynamic forces that accompany breathing we took an open-design approach that leveraged the accessibility of rapid 3D prototyping, Raspberry Pi and Arduino electronics, and open-source repositories for hardware and software design plans. Specific design constraints included the need for 1) a dynamic system that could model diverse breathing patterns, 2) a system scalable for multiple simultaneous experimental replicates, 3) modular in design to allow for different cells and tissue formats to be studied, and 4) suitable for both 2D cell culture and 3D cell and tissue experimentation.

The maximum load and effective stroke length of the CaT stretcher are 500 N and 200 mm, respectively (Table 1 CaT Stretcher Specifications). These specifications ensure that all of cell and tissue culture chips designed in the present manuscript will be amenable to stretching under physiological breathing-like conditions. The combination of operating velocity range and stroke length can effectively model slow (0.2 Hz) shallow (5% strain) breathing and rapid (1.0 Hz) deep (25% strain) breathing on the multiple chip designs presented in the manuscript.

To confirm that the CaT stretcher was performing as the software was programmed to do, we manually measured the stretch lengths with a micrometer through a recording video of 10 min for 1, 2, 5, and 10 mm stretch lengths at 0.2 and 0.4 Hz. An *r* ^2^ value of 0.999 was observed, suggesting that the system was performing stretch at the programmed lengths. An *r* ^2^ value of 1 was observed when a similar analysis was done to confirm the frequency, using 0.2, 0.4, and 1.0 Hz, suggesting the system was perfectly delivering the programmed frequency.

The CaT stretcher is presented with two sizes of aluminum extrusion bars which function as points of attachment for cell and tissue chips. The short (200 mm) and long (400 mm) aluminum rails provide a scalable system for attachment of multiple cell and tissue chips depending on experimental need. As presented with the 3D resin printed mounting bracket (Figures 1C Item # 5), stainless steel mounting pins of 50 mm on the 400 mm rails allows 8 chips to be attached simultaneously (Figures 1C Item # 7). Longer pins of 100 mm length can enable stacking of chambers that can be spaced with M5 nuts of desired thickness to further increase scalability of experimentation (Figure 3).

Traditional 2D submerged monolayer culture is possible with the CaT stretcher in large format surface area (4 cm^2^) and smaller multi-chamber chip designs with a surface area of ∼2 cm^2^ for each chamber (Supplementary Figures S2, S3). The multi-chamber chip was designed in a way that the chambers hold sufficient volume of cell culture media (∼250 µl) enabling downstream analysis of supernatant. The current multi-chamber chip design accommodates 5 chambers per chip but the modularity of the CaT stretcher allows the user to increase or decrease the number of chambers per chip based on the desired application. The CaT stretcher is also compatible with more complex organ-on-chip designs with air-liquid interface culture conditions that enable lung epithelial cell differentiation and polarization (Supplementary Figure S4 **)**. Organ-on-a-chip devices on the CaT stretcher can undergo stretch with and without perfusion. The CaT stretcher does not limit perfusion of fluid or gas/air to the chips. This is an important characteristic of the CaT stretcher for specific applications (e.g., exposure of cells to replicating viruses in a biosafety cabinet). Hydrogels and live tissue strips, which both represent 3D model systems of the *in situ* microenvironment are also amenable to experimentation within the CaT stretcher (Supplementary Figures S5, S6).

The presented CaT stretcher and chip designs satisfy the *a priori* defined constraints and are open-access to ensure uptake is possible by the widest available audience. (Figures 2; Supplementary Video S1).

### 3.2 Polydimethylsiloxane chip coating for submerged monolayer stretch experimentation

The decision to use PDMS as a material for chip design required a consideration for optimization of the surface, as PDMS is hydrophobic and does not contain biochemical cues present in the *in situ* lung^49^ *.* We therefore investigated different coating strategies to find a surface modification for submerged cell cultures that enabled cell adherence and viability under resting and stretch conditions. These strategies are divided to two groups: 1) Physical adsorption of collagen (COL) and fibronectin (FN) or gelatin (degraded form of collagen) onto PDMS surfaces or 2) bonding of these proteins onto PDMS surfaces using a PDA coating and adhesion promoter.

**FIGURE 5:**
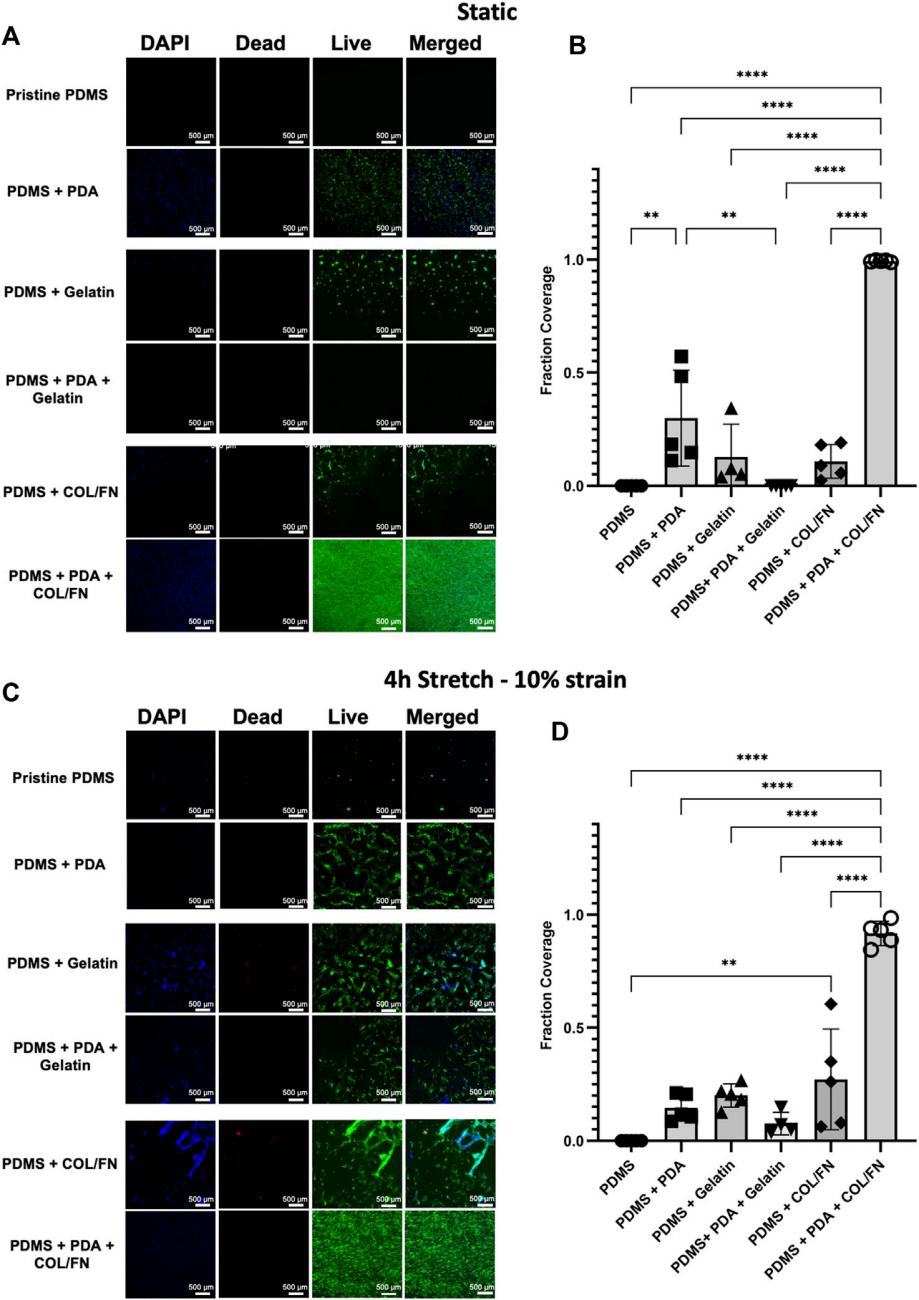
PDMS coating strategies for increasing HLFC adherence and proliferation under static and stretch conditions. **(A–B)** Static experimental conditions. **(C–D)** Stretch—10% mechanical strain for 4 h. **(A,C)** DAPI (blue) and live (green)/dead (red) imaging of primary HLFC under diverse PDMS coating conditions for **(A)** static control and **(C)** stretch. Pristine PDMS is uncoated control. See Materials and Methods for details on PDA, gelatin, and COL/FN coatings. Representative images of regions of interest from a submerged monolayer culture multichannel chip (see Supplementary Figure S3) are presented. **(B,D)** Quantification of cell culture surface area covered by live cells under each coating condition. **p < 0.01, ****p < 0.0001.

HLFCs were seeded on all surface areas at the same cell density (∼1.5 ˣ 10^4^ cells/cm^2^) and monitored for cell adherence and growth. Once a single experimental surface area reached confluence, the mechanical stretch was initiated. This decision to start experimentation upon confluence of the fastest growing experimental condition was made due to the observation that some conditions (e.g., PDMS with no coatings) failed to promote cell adherence and proliferation despite the same seeding density used in other experimental conditions. HLFCs were subjected to control condition (no stretch) or stretch (10% strain) for 4 h followed by analysis of cell viability with a LIVE/DEAD™ Cell Imaging (Figure 5).

The fraction coverage of cells for all conditions was quantified in ImageJ software as shown in Figure 5. The adhesion and growth of HLFCs on PDMS surfaces were minimum confirming that pristine PDMS is not an optimal surface for cell growth. This observation agreed with our previous study showing that pristine PDMS surfaces were not optimal for culturing human primary airway epithelial cells (Dabaghi et al., 2021b). Physical adsorption of gelatin and COL/FN partially improved cell attachment but did not facilitate full cell coverage. The data suggest that physical adsorption of gelatin and COL/FN may not be uniform or permanent. In contrast, PDA coating showed significant improvement in cell adhesion and growth, with further addition of COL/FN coatings being superior under both static and stretch conditions (Figure 5). Except for PDA + COL/FN coating, none of the coatings reached a confluency greater than 50% under static and stretch conditions. PDA + COL/FN coating was the only condition with consistent cell coverage before and after applying stretch. Both quantitative and qualitative data suggested that PDA + COL/FN coating resulted in higher cell attachment and proliferation.

**FIGURE 6:**
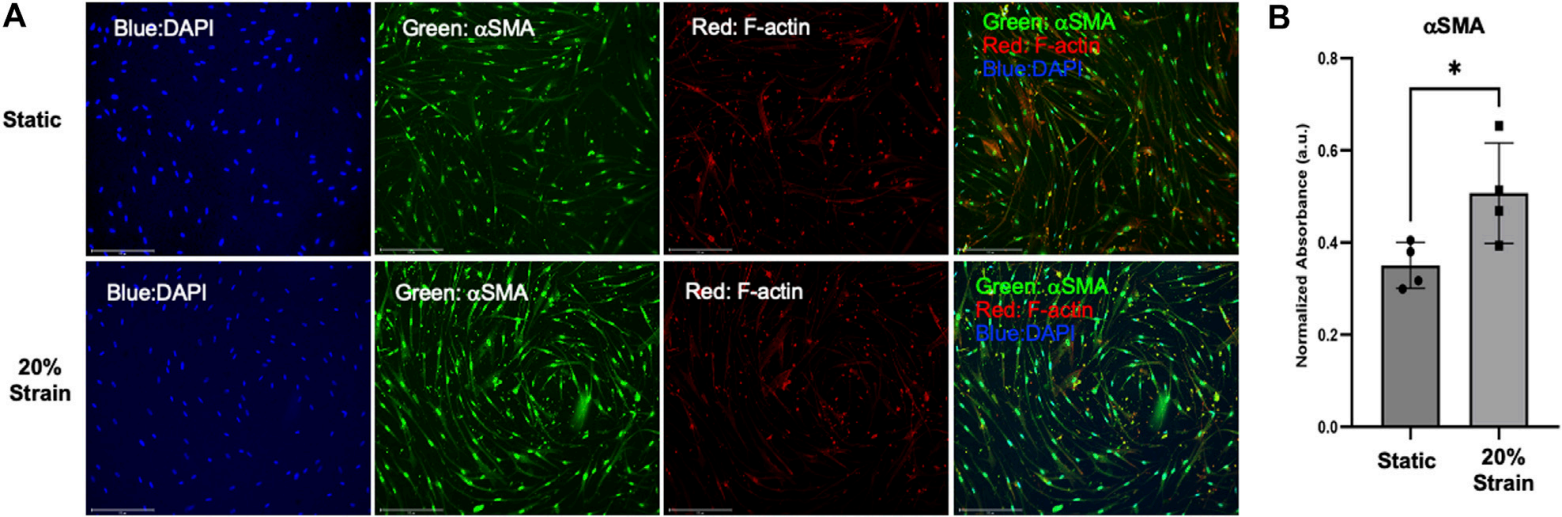
αSMA protein expression is upregulated in HLFC cultured under submerged monolayer culture conditions following stretch. **(A)** Representative wide-field fluorescent microscopy with DAPI (blue), αSMA (green), F-actin (red) and merged images for static control and 20% strain. **(B)** Quantification of αSMA expression as a measure of fluorescent signal (green channel) normalized to total cell number (DAPI nuclei). * = *p* < 0.05.

Following optimization of the coating strategy for primary HLFCs, we initiated stretch for 4 h and quantified alpha-smooth muscle actin (αSMA) as a biological readout of myofibroblast transformation under strain and control of YAP (Piersma et al., 2015). αSMA protein was measured by fluorescence intensity and observed to be elevated in primary HLFCs cultured on PDA + COL/FN with stretch, relative to non-stretch control (Figure 6).

### 3.3 Viability of cells within hydrogel under static and stretch conditions

**FIGURE 7:**
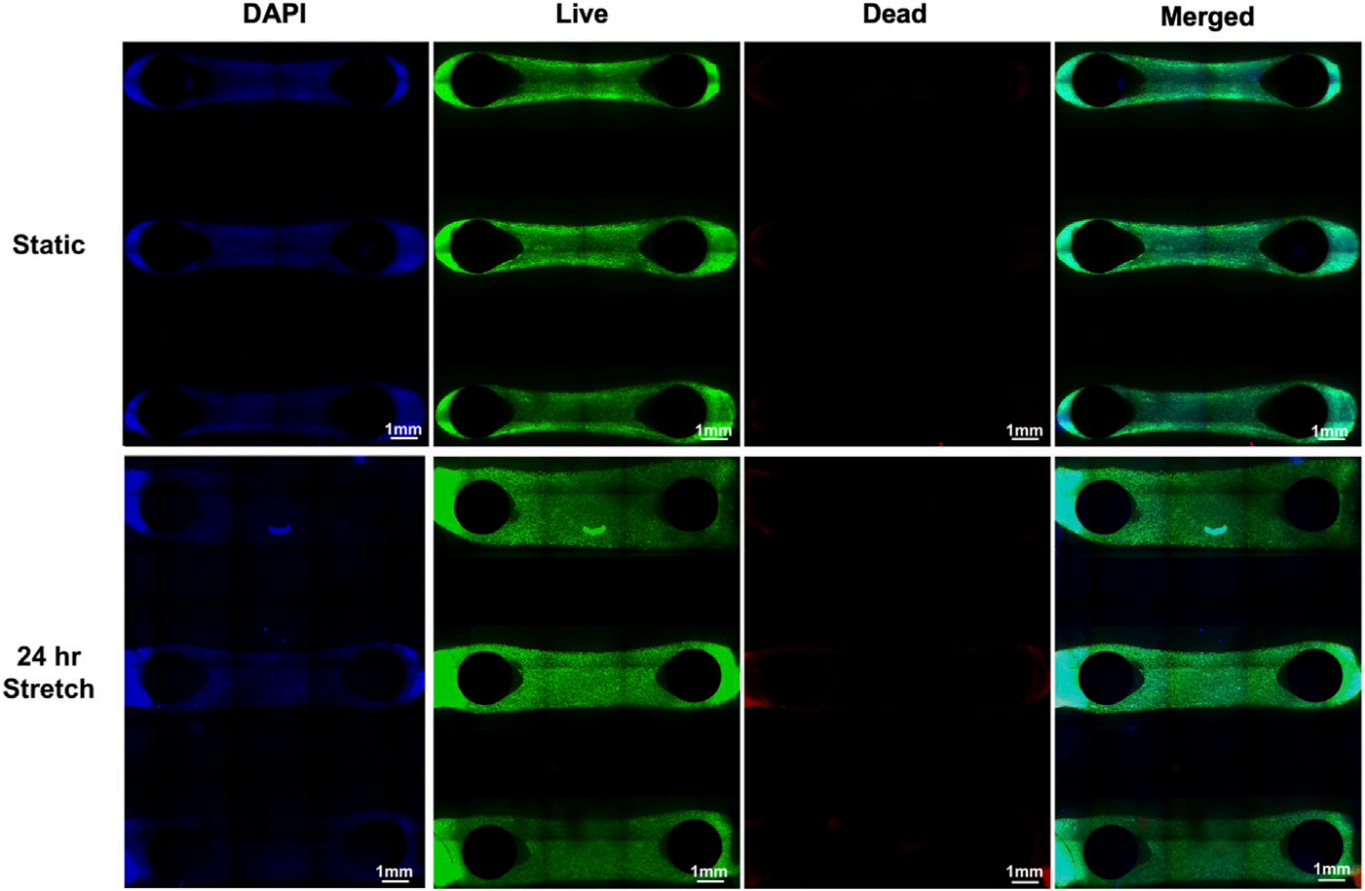
3D hydrogel pillar chips with embedded HLFC tissues for modeling the microenvironment under static and stretch conditions. DAPI (blue) and live (green)/dead (red) imaging of HLFC grown in 3D hydrogel tissue structures (see Materials and Materials for details). Stretch of 10% mechanical strain was provided for 24 h under conditions of 37°C in a cell culture incubator followed by live/dead cell imaging and microscopy. Qualitative images are shown as only sparse dead (red) cells were detected and quantification revealed no differences between static and stretch conditions.

A microfluidic chip with two pillars was designed and fabricated to stretch cells in a 3D environment (Supplementary Figure S5). This design leveraged the natural contraction of HLFCs in a 3D ECM environment that we have previously reported (Dabaghi et al., 2021a) and led to the formation of dumbbell-shaped hydrogels wrapped around pillars that were constrained of any free movement within the PDMS chip. A greater cell density was observed behind the pillars as the hydrogel material contracts with the embedded fibroblasts. After forming constrained hydrogels, the viability of cells with and without stretch using the CaT stretcher was evaluated using the described LIVE/DEAD™ Cell Imaging assay (Figure 7). Qualitative visual analysis of HLFCs viability in constrained hydrogels without and with 24 h stretch with the CaT stretcher showed high cell viability and negligible cell death. These results demonstrate that the CaT stretcher and 3D hydrogel chip design is amenable to modeling HLFCs under control static and experimental stretch conditions.

### 3.4 α-smooth muscle actin expression in primary human lung fibroblast cells in hydrogels following stretch

To further demonstrate the applicability of the CaT stretcher in modeling mechanosensitive processes beyond submerged monolayer cultures, we characterized the expression αSMA in HLFCs under static and stretch conditions in the 3D hydrogel chip design using widefield and confocal fluorescent microscopy.

**FIGURE 8:**
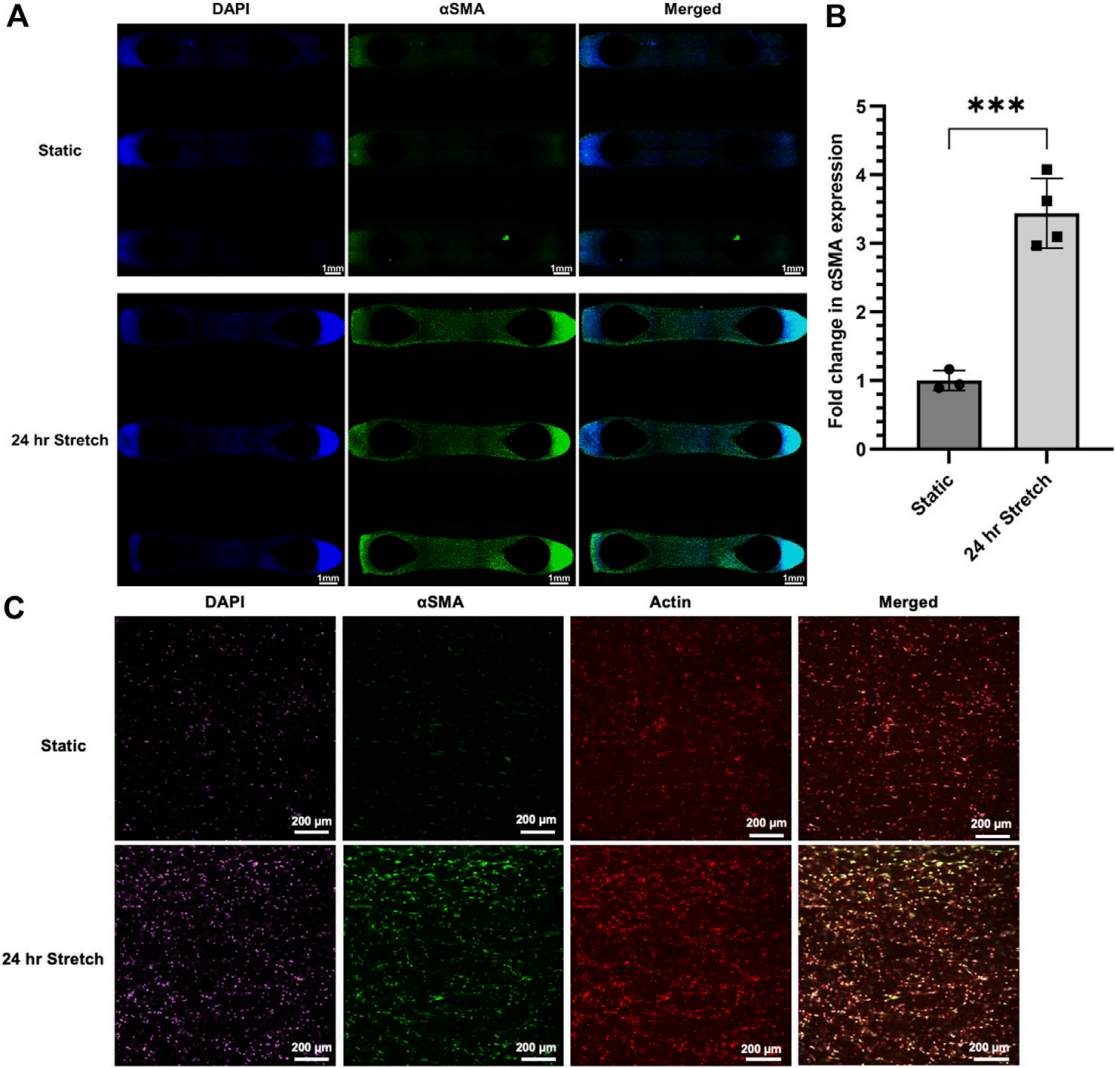
αSMA protein expression is upregulated in HLFC embedded in 3D hydrogels following stretch. **(A)** Wide-field fluorescent microscopy with DAPI (blue), αSMA (green), and merged images presented as tiled images of the entire hydrogel culture. Stretch was induced by 10% mechanical strain for 24 h under conditions of 37°C in a cell culture incubator. Control hydrogels were treated identical except without stretch. **(B)** Quantification of αSMA expression as a measure of fluorescent signal (green channel) normalized to static control condition. **(C)** Confocal fluorescent microscopy of select regions of interest from the above hydrogel experiments with DAPI (purple), αSMA (green), actin (red), and merged images. Qualitative increases in αSMA (green) were shared between wide-field and confocal microscopy. *** = *p* < 0.001.

Continuous stretch for 24 h induced strong qualitative protein expression of αSMA in hydrogel samples (Figure 8A). Quantification of αSMA staining under wide-field fluorescent microscopy of the entire hydrogel sample confirmed qualitative observations with an ∼3 fold increase in protein expression induced by stretch as measured by fluorescence intensity (Figure 8B). Confocal microscopy was used to provide greater resolution images. This secondary microscopy approach confirmed the quantification results and revealed spindle-shaped αSMA expressing HLFCs, which is suggestive of a transition to a myofibroblast phenotype (Figure 8C). The magnitude and resolution of images acquired did not provide the ability to assess cell alignment.

### 3.5 α-smooth muscle actin expression in live lung tissue slices following stretch

**FIGURE 9:**
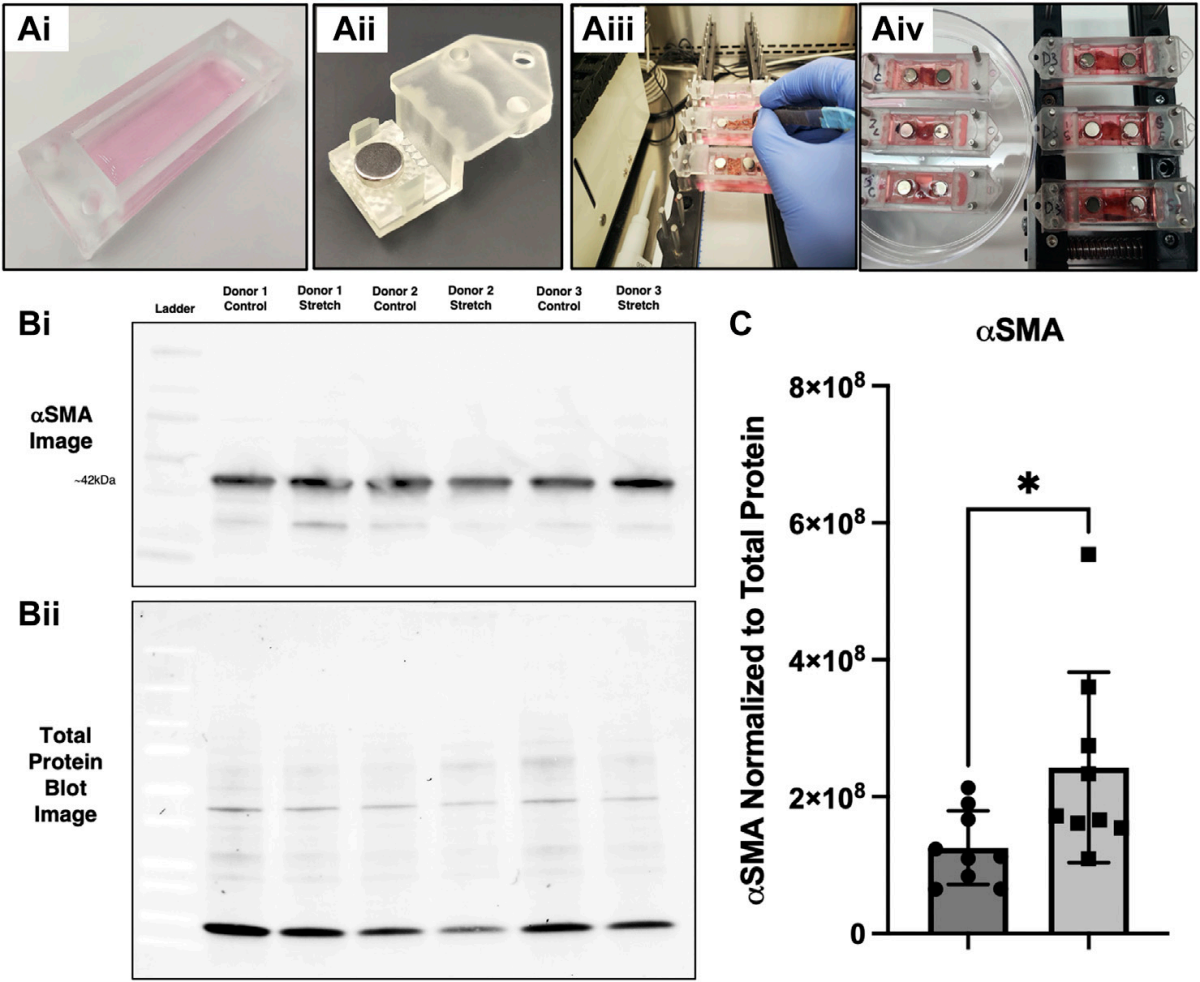
αSMA protein expression is upregulated in live lung slices following stretch. **(Ai–Aiv)** Experimental setup with tissue clamp chambers, placement of chambers on the CaT stretcher, and setting up the experiment with controls (no stretch) and experimental replicates (20% strain). **(Bi)** Representative αSMA immunoblot with signal from 3 distinct donor samples with strain and control samples run in adjacent lanes. **(Bii)** Representative total protein image taken from the same blot. **(C)** Quantification of αSMA expression (*n* = 9 replicates from 5 donors), * = *p* < 0.05.

Live lung slices represent an advanced model that provides the *in situ* microenvironment, cell populations, and spatial distribution, making them a valuable tool for lung research. Lung slices have been studied under static and dynamic conditions (Dassow et al., 2010; Mondoñedo et al., 2020). Using our tissue clamp chamber system, we performed a series of experiments with resected lung tissue from routine surgical procedures (Figures 9Ai,Aii). The lung slices were placed in the chambers with controls being treated identical to stretch, except for the absence of stretch (Figures 9Aiii,Aiv). The CaT stretcher mechanical device with the tissue clamp chips containing lung slices was placed in an incubator for 24 h. Following 24 h, the region of tissue that was stretched was dissected away from the areas that were clamped to isolate only strained tissue. Protein isolation and immunoblotting using Bio-Rad TGX-Stain-free gels with total protein normalization methods for quantification of αSMA (Gürtler et al., 2013) (Figures 9Bi,Bii). αSMA protein was elevated in lung slices following 24 h stretch when normalized to total protein content (Figure 9C).

## 4 Discussion

The CaT stretcher was built off a foundation of open-design and modularity for end-user applications beyond our original intentions. This overarching philosophy would ensure that the CaT stretcher could evolve with a given research groups directions and questions. Our design constraints included the need for 1) a dynamic system that could mimic normal and diseased breathing patterns, 2) scalable to enable efficient experimentation with simultaneous control and treatment groups possible, 3) modular in design to allow for different cells and tissues to be studied, and 4) suitable for both 2D and 3D experimentation. As an environmental constraint, the device needed to be amenable for use in 1) benchtop, 2) biosafety cabinets and 3) incubators. Extending beyond the device, the mechanism for connecting cell and tissue samples to the device would need to enable access to the samples for outcomes frequently used in the biomedical research environment, including, but not limited to microscopy, soluble mediator measurement, and gene and protein expression. Lastly, to increase the uptake of the device within the community and future adaptability, the system needed to be open-source and built with economically feasible and available components. The net result of these constraints is the programmable CaT stretcher and a suite of PDMS based cell and tissue chip designs that are consistent with commercially available devices and consumables. We anticipate that the CaT stretcher will become a foundation for greater exploration into the dynamic lung microenvironment through direct implementation by other research groups or custom iterations on our open-design.

Our technical report must be accompanied by a discussion on the CaT stretcher limitations. Firstly, the uniaxial stretch delivered to cell and tissues within our device may not reflect multi-axial strain experienced *in vivo*. Second, our modular platform that can be used with submerged monolayer, 3D hydrogels, organ-on-chips, and live tissue slices, requires research labs to have specialized fabrication infrastructure for chambers and relies on PDMS as a material. The 3D hydrogel chamber has a specific limitation resulting from different patterns of cell arrangement around the pillars. The tissue clamp chamber has a limitation that only the tissue between clamps undergoes strain while the clamped regions are static. Third, although open-source, the CaT stretcher requires mechanical, electrical, and fabrication engineering experience. We describe these limitations in greater detail below.

The CaT stretcher and chips were designed to enable studies focused on the different aspects of the cell and tissue microenvironment. The role of the microenvironment on lung cell biology grew from an understanding of lung mechanics (Wiggs et al., 1990; McGowan, 1992; Suki et al., 2005; Roan and Waters, 2011) and clinical observations that aberrant strain could induce lung injury as observed in ventilator-induced lung injury (RG et al., 2000). Basic and translational research grounded in this clinical reality has defined the diversity of lung cell types that are impacted by strain and stress experienced in the cell and tissue microenvironment. Early studies with dynamic *in vitro* model systems demonstrated that strain induced surfactant production from type II alveolar epithelial cells (Wirtz and Dobbs, 1990), changes in airway smooth muscle contractility and tone (Shkumatov et al., 2015), and fibroblast-to-myofibroblast transformation (Wipff et al., 2007). Under specific conditions, strain experienced by lung cells is associated with release of danger associated molecular patterns that can induce innate immune responses. Specifically, ATP can be released from multiple lung cells including alveolar epithelial cells, airway smooth muscle cells, and immune cells (Ramsingh et al., 2011; Murata et al., 2014; Takahara et al., 2014; Tan et al., 2020). Collectively, these data provide an important link between strain from the lung microenvironment and clinical pathologies observed during ventilation and infection, made more relevant during the COVID-19 pandemic. The tunable nature of the CaT stretcher and chips provides an easy to use, open-source platform for studying the impact of strain on lung cells and tissues, with immediate applications in further defining the links between the dynamic lung microenvironment and immune responses important in healthy and pathologic lung settings.

A dominant cue that we sought to integrate into the CaT stretcher designs and downstream applications were the biochemical cues communicated from the ECM (Zhou et al., 2018). Lung ECM is critical in regulating cell differentiation and phenotype during development, homeostasis, and responses injury (McGowan, 1992; Sava et al., 2017). Biochemical cues from the ECM are translated to intracellular responses *via* integrins that function to connect the external cell and tissue microenvironment with the internal transcriptional control of a cell (Hynes, 2002; Bachmann et al., 2019). Integrins are composed of combinations of α and β subunits and form dimers at the cell surface and connect with the actin cytoskeleton at focal adhesions. Signaling downstream of integrins coordinates intracellular cascades that involve nuclear translocation of two known mechanosensitive transcription factors, YAP and TAZ (Dupont et al., 2011; Brusatin et al., 2018). The biochemical cues provided by the lung ECM are variable with age (Gilpin et al., 2017) and the presence of underlying pathology (Booth et al., 2012; Parker et al., 2014; Wagner et al., 2014). Using static model systems of decellularized lung, biochemical cues have been demonstrated to impact basal cell (Gilpin et al., 2017) and fibroblast to myofibroblast transition (Parker et al., 2014). Importantly, tissue studies utilizing healthy and fibrotic lung samples under dynamic stretch conditions further reveal biological processes important in lung pathology not observed under static conditions (Froese et al., 2016; Shimbori et al., 2019). A proposed mechanism defining the stretch-induced activation of biological processes only present in fibrotic lung samples involves the biochemical cues from the ECM, integrin signaling, and intracellular cytoskeleton rearrangement that creates stiff cells responsive to TGF-β1 signaling (Wipff et al., 2007; Hinz and Suki, 2016). Ultimately, both static and dynamic models have emphasized the importance of integrating biochemical cues from the ECM into model systems and where possible, mechanical forces that accompany simulation of breathing. In the present paper, we have developed CaT stretcher chips amenable to coating with different ECM components, a hydrogel pillar chip that incorporates fibroblasts cultured in ECM in 3D, and a chip design for live tissue stretching that relies on *in situ* ECM present in the sample of study. Our chip designs will have broad applicability for studying biochemical cues important in the lung cell and tissue microenvironment for diverse *in vitro* and *ex vivo* models and our preliminary studies validate exploration of fibroblast biology under dynamic conditions.

Conventional lung cell culture models have frequently relied on submerged monolayer culture conditions, which fail to adequately incorporate the 3D microenvironment experienced by lung cells. Hydrogels represent an experimental approach that overcomes limitations of submerged monolayer culture systems by providing 3D environmental cues in the form of biochemical, topographical, and mechanical signals (Smithmyer et al., 2019; De Hilster et al., 2020; Dabaghi et al., 2021a). Hydrogels are softer than tissue culture plastic, which has important consequences on lung cell biology mediated in part through the mechanosensing transcription factors, YAP and TAZ (Marinković et al., 2013; Liu et al., 2015). Furthermore, hydrogels are tunable in stiffness and can be made from a variety of input materials including single or complex ECM components from human or non-human sources (Kopeček, 2007; Kim et al., 2020). Human lung decellularization protocols have provided a means to use lung material donated for medical research to isolate ECM and create hydrogels that resemble the relative composition of the *in situ* lung (Smithmyer et al., 2019; De Hilster et al., 2020; Dabaghi et al., 2021a). Mechanical testing of human lung derived ECM hydrogels demonstrates similar properties to native lung tissue (De Hilster et al., 2020), which is consistent with previous reports that show preservation of Young’s Modulus between native and decellularized tissue in both health and diseased tissue samples (Booth et al., 2012). The ability of 3D hydrogels to mimic the *in situ* cell and tissue microenvironment has been validated using clinically used antifibrotics in a hydrogel microtissue system to prevent and reverse lung myofibroblast transformation (Asmani et al., 2018), demonstrating the potential promise for hydrogel approaches in drug development pipelines. Preparation of hydrogels for *in vitro* models may result in variability between experiments due to heterogenous distribution of ECM components and cells within the structure (Asmani et al., 2018). Hydrogel chip designs can also enable introduction of desired variability. Our hydrogel chip design presents distinct tissue microenvironments—one between the pillars which is exposed to stretch and another behind the pillars which is exposed to the presence of an obstruction. Our design enables simultaneous exploration of mechanotransduction processes in two distinct microenvironments, with both showing induction of αSMA in primary lung fibroblasts following stretch. In closing, the CaT stretcher has been designed and validated to be amenable to 3D hydrogel applications and will enable both basic science discovery studies and translational drug screening applications.

Commercial cell and tissue stretching devices are available and have been used reported on extensively in the literature for lung biology and beyond. *In vitro* lung devices that introduce mechanical forces and exposures have recently been reviewed extensively (Nossa et al., 2021). Platforms include those manufactured by FlexCell, STREX, ShellpaPro, CellScale, and Emulate. Like the CaT stretcher, these systems can be used for more than just lung cells and tissues. Commercially available platforms offer opportunities to apply mechanical forces through stretching while also introducing topography, stiffness, and biochemical cues through cell culture coatings. These devices offer programmability for stretching through easy-to-use interfaces that vary in connectivity to computer controllers, ranging from Wifi/Bluetooth to hard wired. A trade-off with commercially available devices is their intrinsic closed hardware/software design that may constrain iterative experimentation and upfront costs for hardware and dependency on consumables from the original manufacturer. The presence of these tools on the market validates an unmet need that is also frequently satisfied by custom designs that are more modular in design and have the potential for bespoke applications at reduced costs (Huang et al., 2010; Seriani et al., 2016; Mayer et al., 2019; Al-Maslamani et al., 2021; Cei et al., 2021; Correia Carreira et al., 2021; Daulagala et al., 2021; Doryab et al., 2021). In-house designs are frequently open in nature and becoming more approachable due to the availability of rapid prototyping and 3D printing (Al-Maslamani et al., 2021). Open-source uniaxial and biaxial stretchers with variations in throughput capacity have been reported and demonstrate the diversity in dynamic cell and tissue platforms for studying mechanotransduction (Kurata et al., 2019; Shiwarski et al., 2020; Kah et al., 2021). Researchers interested in introducing stretch to their experiments may require prioritization of one parameter of a given system (e.g., biaxial) (Shiwarski et al., 2020) over another (e.g., throughput) (Kah et al., 2021). Integration of heating to maintain a desired temperature (Shiwarski et al., 2020) is a feature that may not be available in other open-source designs (Kurata et al., 2019; Kah et al., 2021), although the ability to place an apparatus in a humidified incubator (e.g., our CaT stretcher) may circumvent the need for heating. Another important consideration is the ability to perform microscopy in real-time, which some systems have developed (Shiwarski et al., 2020). Even LEGO^®^ has recently been used in an open-design (Boulter et al., 2020). We present a complete open-source design for the CaT stretcher for full access to all considerations needed to replicate the instrument and leverage for further developments and experimentation. Not all research groups will be comfortable building their own devices, even from well-documented open-source designs, and the market will likely benefit from a continued access to turn-key solutions from commercial providers and publicly accessible designs that are modular and free. Iterations on tool and component designs in the academic environment may represent future product offerings for commercial providers, increasing the global accessibility to dynamic cell and tissue modeling devices/infrastructure.

The CaT stretcher was designed with a priority on modularity and scalability to provide multiple research questions to be answered with a single device that can perform static or dynamic stretch. Our design enables traditional 2D submerged monolayer cultures, organ-on-chip devices with air-liquid interface capacity, 3D hydrogels, and live tissues. A trade-off of our design is a system of reduced complexity that stretches only in a uniaxial direction, in contrast to commercially available and custom designs that are biaxial, multiaxial, or radial stretchers. Any cell and tissue stretching device that is motor driven may induce mechanical forces on cells that are distinct from those experienced *in situ,* where transpulmonary pressure gradients are what define cell and tissue stretching during breathing (Dong et al., 2022). Both negative and positive pressure driven cell and tissue stretching systems are available (Breen, 2000; Huh et al., 2010; Blaauboer et al., 2011; Stucki et al., 2015). Divergent lung resistance and elastance values have been observed between negative and positive pressure driven analyses of lung mechanics (Dong et al., 2022), suggesting that conclusions from a single dynamic model system should be cautious and explored in diverse model systems, a process made possible with the modularity of the CaT stretcher.

The pioneering lung-on-chip model was developed with a pressure driven vacuum mechanism for cell stretching, that pulled uniaxially on a flexible membrane containing epithelial cells on the apical surface and endothelial cells on the basal surface (Huh et al., 2010), which has resulted in a commercial product made by Emulate (Si et al., 2021). Alternate lung on chip devices have been developed that share the air-liquid interface design and are amenable to introduction of airflow on the apical side and fluid flow on the basal side (Stucki et al., 2015; Benam et al., 2016; Jain et al., 2018). To ensure modularity and flexibility for use of organ-on-chip approaches, the CaT stretcher was designed to accommodate ALI-culture with ability for apical and basal perfusion. Pulsatile flow from peristaltic pumps can be generated in organ on chip devices on the CaT to introduce mechanical strain in the absence of vacuum driven pressure changes. In addition, the modularity of the CaT stretcher has the capacity to combine different forms of cell stretching for organ-on-a-chip devices, where mechanical stretch and pressure-driven vacuum stretch can be both be applied to cells.

The *ex vivo* modeling of the *in situ* lung environment may be most accurately reflected using live lung tissue donated for research purposes. The precision cut lung slice (PCLS) method has emerged as a compelling approach to maintain *in situ* microenvironments, the spatial distribution of cells, and the relative number of cells, in a scalable system that can generate multiple samples from a single specimen (Jiang et al., 2020). The PCLS approach can compare specimens from healthy controls and those with clinically diagnosed pathology, enabling direct *ex vivo* comparison of complex multicellular lung samples. Importantly, the PCLS approach can be studied under static and dynamic conditions, with approaches for biaxial distention possible using mechanical and vacuum driven stretch (Dassow et al., 2010; Mondoñedo et al., 2020). The combination of live lung tissue in PCLS or similar dissected formats with dynamic *ex vivo* platforms is important, as processes relevant in disease progression may only reveal themselves under stretch. Our group has demonstrated that stretch augments fibrotic lung tissue slice release of TGF-β1, potentially feeding forward the cycle of fibrotic remodeling (Froese et al., 2016). These observations were dependent on the combination of live lung tissue in the native lung ECM environment and mechanical stretch. The CaT stretcher tissue clamps have been designed to accommodate live lung tissue in PCLS or dissected formats (Scholze et al., 2018), with scalability for multiple samples stretched simultaneously, opening up the possibility of increased throughput with complex *ex vivo* model systems.

The most frequently used material for cell and tissue chip stretching devices is PDMS. PDMS is an ideal material as it is non-cytotoxic, optically clear, has mechanical properties similar to biological tissues, its mechanical properties can be modified based on polymer and curing agent ratio, and can be cast into molds created by standard 3D resin printers. PDMS also has limitations, including absorbent properties that may impact the ability to detect metabolites or administer drugs accurately. For a submerged monolayer cell culture system or certain organ-on-chip designs, the use of PDMS as a growth surface may require coating with additional materials to more closely mimic the *in situ* topographical and biochemical microenvironment provided by ECM (Dabaghi et al., 2021a; Dabaghi et al., 2021b). The PDMS surface is hydrophobic in nature and is not conducive to cell attachment and proliferation, meaning a proper surface treatment is needed to enhance these properties. Air or oxygen plasma is a common technique to alter the hydrophobicity of PDMS surfaces by oxidizing them. This method is prone to hydrophobic recovery over time and may be not effective for all cell types or experiments accompanied by mechanical stretch. An alternative approach is to tailor PDMS surfaces with molecules that improve the attachment of cells. ECM-based proteins have been shown as the best biochemical cues for increasing cell adhesion. To overcome the limitation of PDMS surface properties, coating can be performed through a variety of methods using collagen, fibronectin, gelatin, or PDA (Shakeri et al., 2021). These molecules can be attached to PDMS surfaces by physical adsorption or chemical bonding. Although PDMS has high affinity to physically adsorb proteins and it is an easy approach to enhance cell adhesion, it might not be suitable for dynamic cell culture system that are continuously subjected external stresses such as stretch. Therefore, we explored a few different common coating strategies aiming for the one that is stable for static and dynamic cell culture experiments. The requirement for PDMS coating may be limited to submerged monolayer cultures and organ-on-chip devices with PDMS membranes, as 3D cultures or live tissues that are held in solution within PDMS chips will not be influenced by the topography or biochemical cues of the chip walls and surface area. Overall, PDMS represents an accessible, industry standard material that can be customized by research groups to create cell and tissue microenvironments that are able to be stretched to model dynamic living conditions. In this exploration, we ensured that the optimum coating strategy has the potential for mobilizing various ECM proteins on the PDMS surface. For instance, our group developed a decellularization process for human lung tissues so that the decellularized tissue can be digested for different applications, such as coating various 2D monolayer cell cultures (Dabaghi et al., 2021a). The PDA coating approach will enable us to coat PDMS-based chips with decellularized lung tissue materials from healthy subjects and those with lung disease for studying the impact of different biochemical cues on cells in combination with mechanical stretch.

The lung is a continuously dynamic organ that is responsible for 10000 L of ventilation each and every day in humans. Using dynamic *in vitro* model systems, important lung cell and tissue processes that are impacted by the dynamic microenvironment have already been defined and include lung development, immunity, repair processes, and aging. Indeed, there likely is no lung process that is not impacted indirectly or directly by mechanical forces exerted by the microenvironment. In the present manuscript we present the development and validation of the open-access CaT stretcher to enable exploration into lung processes in health and disease under dynamic conditions in a scalable, modular, and programmable system.

## Data Availability

The original contributions presented in the study are publicly available. This data can be found here: https://github.com/crystalliu314/CaT_Stretcher.
