# Supplementary figures and images for: The CaT stretcher: An open-source system for delivering uniaxial strain to cells and tissues (CaT)
Source: Front Bioeng Biotechnol. 2022 Oct 18;10:959335. doi: 10.3389/fbioe.2022.959335 (PMC9622803; doi:10.3389/fbioe.2022.959335)

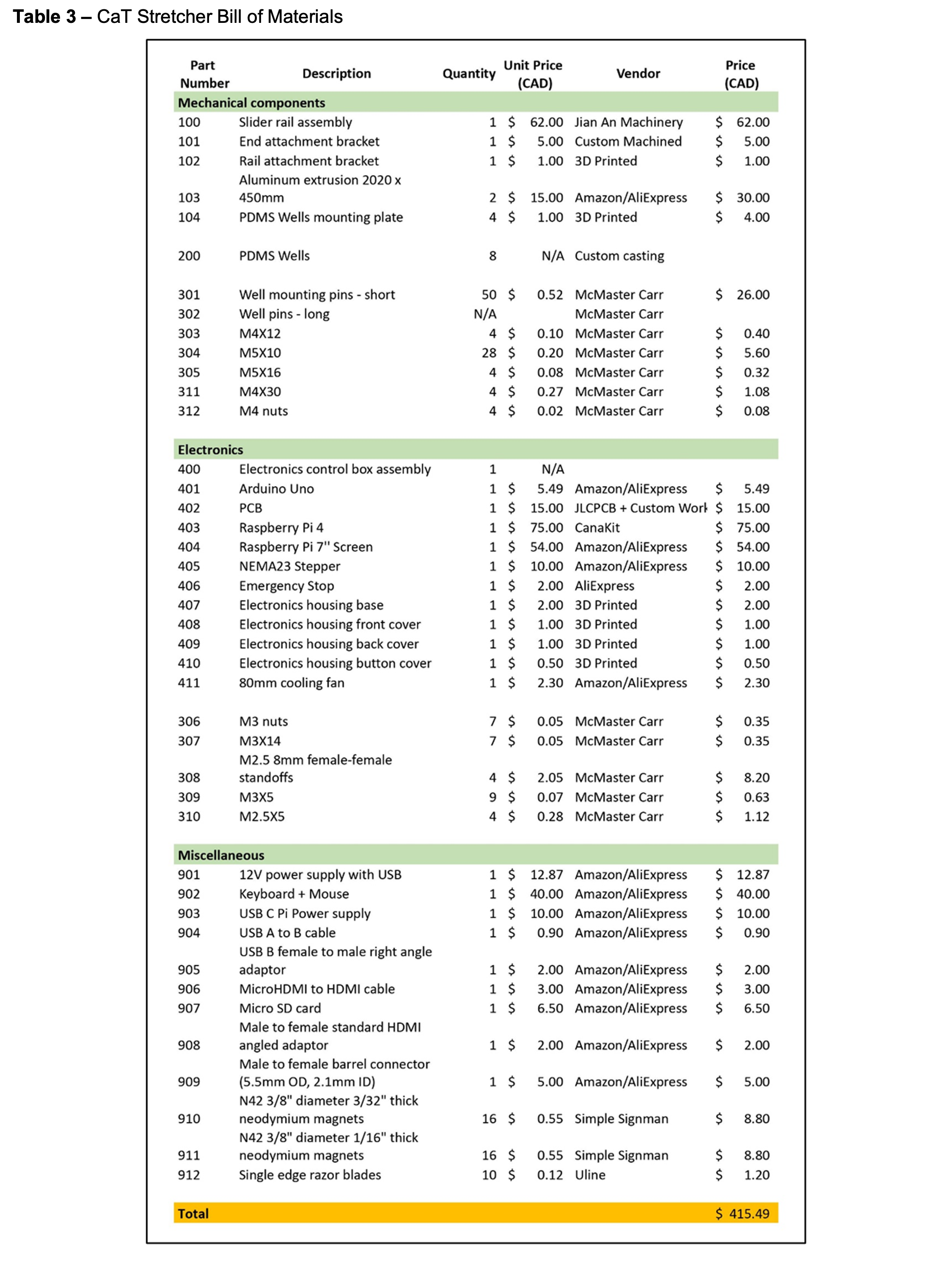

Supplement: Supplementary file 1 [file Image3.TIFF]

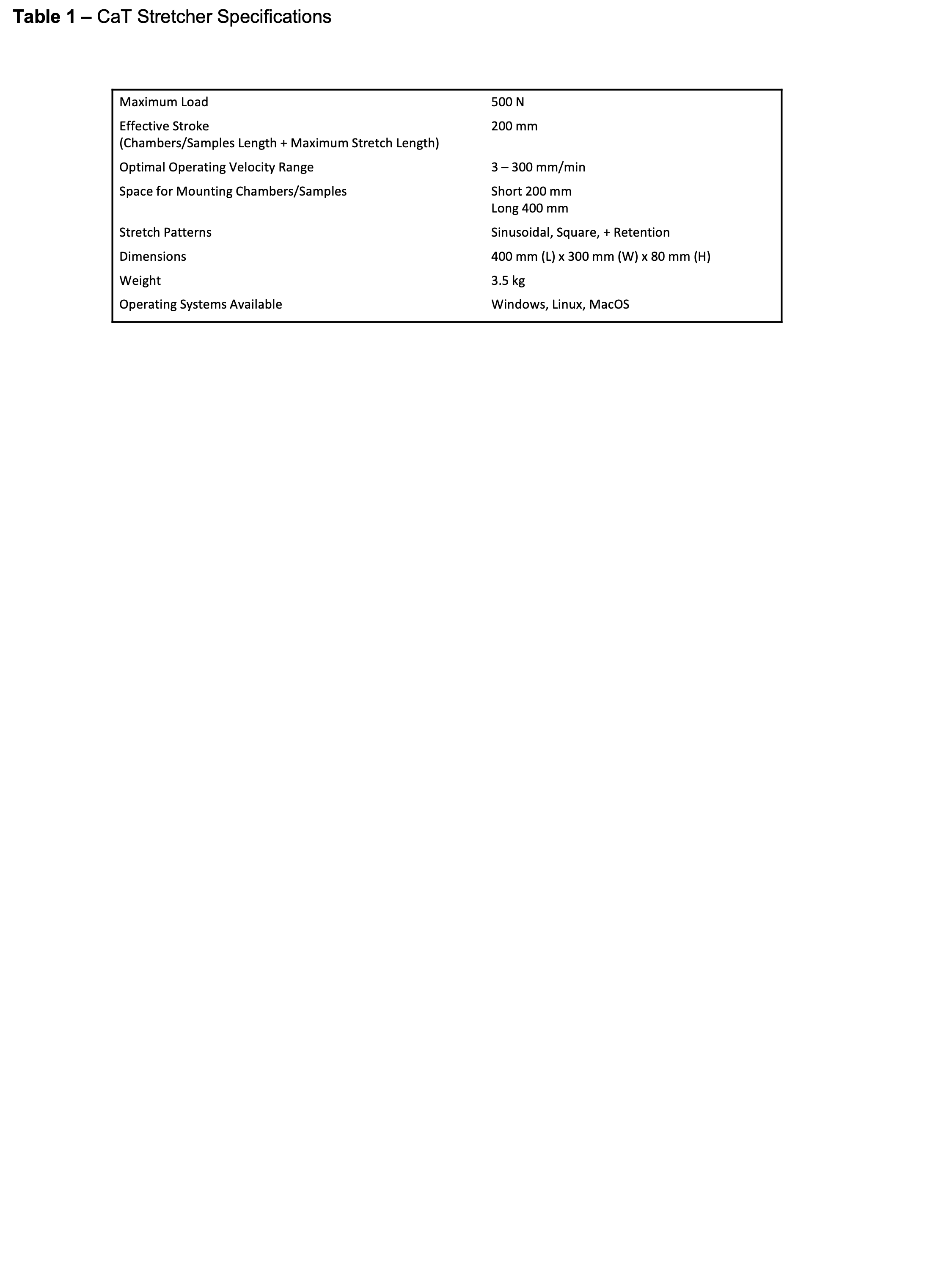

Supplement: Supplementary file 2 [file Image1.TIFF]

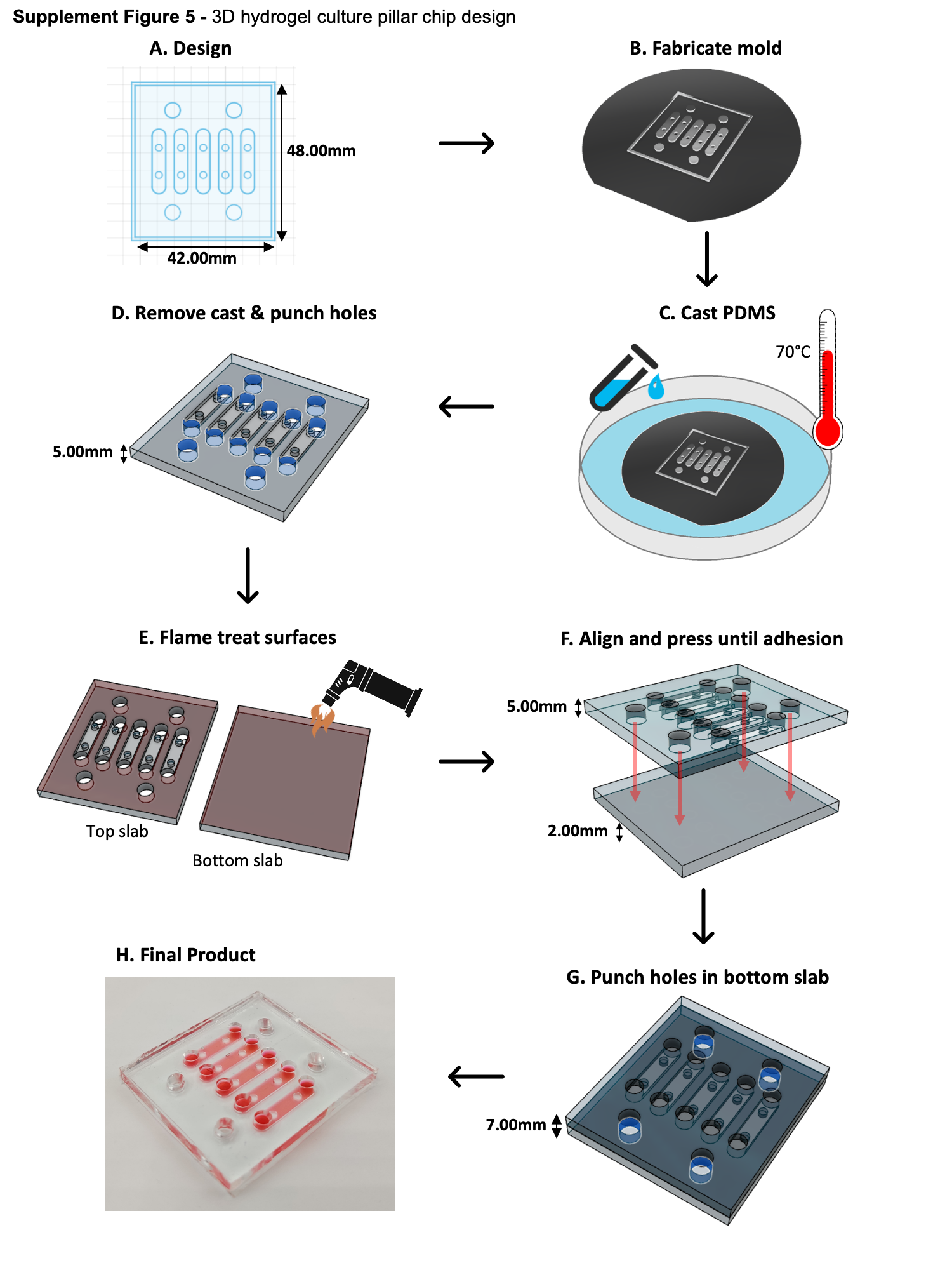

Supplement: Supplementary file 3 [file Image9.TIFF]

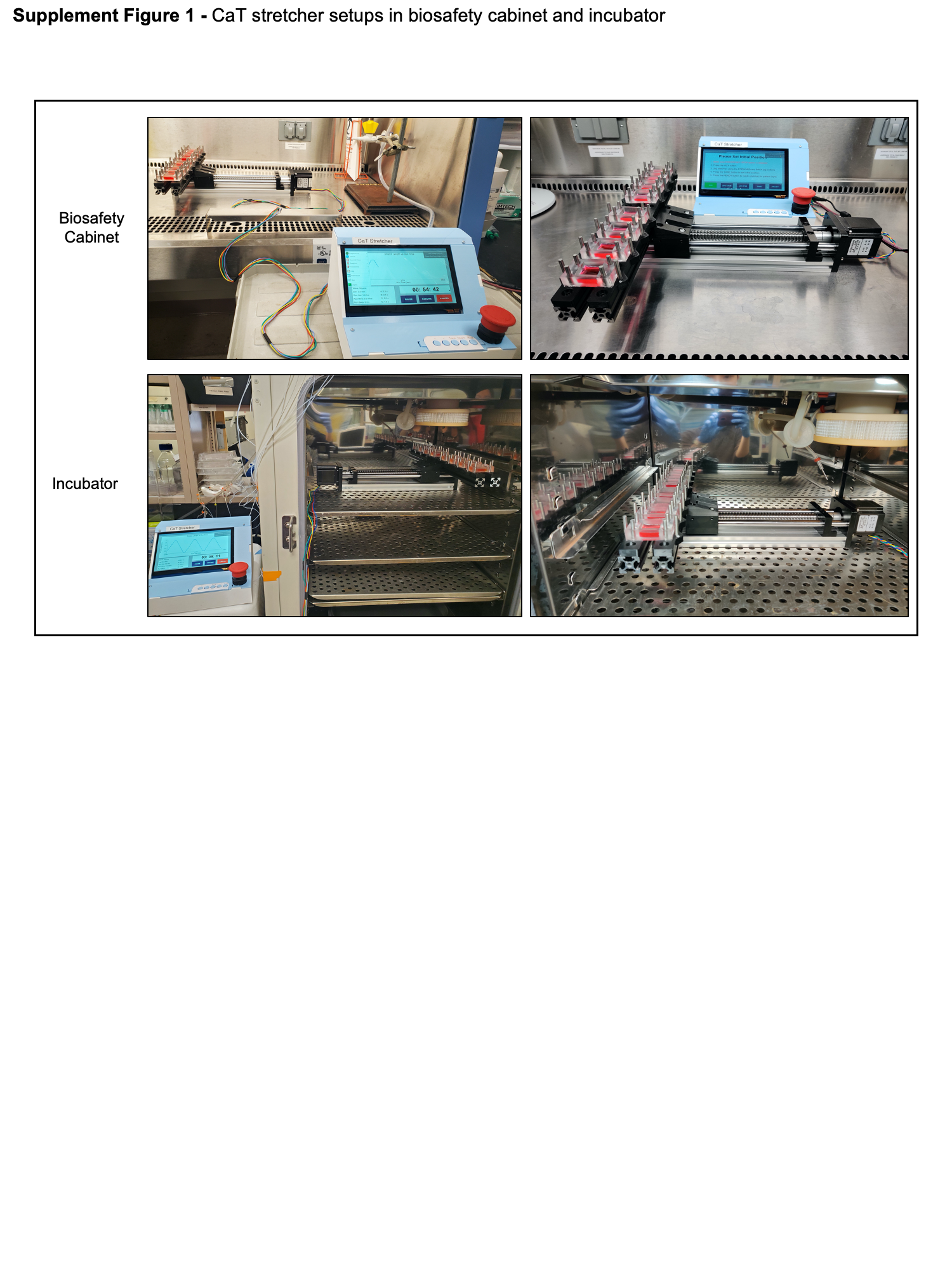

Supplement: Supplementary file 4 [file Image5.TIFF]

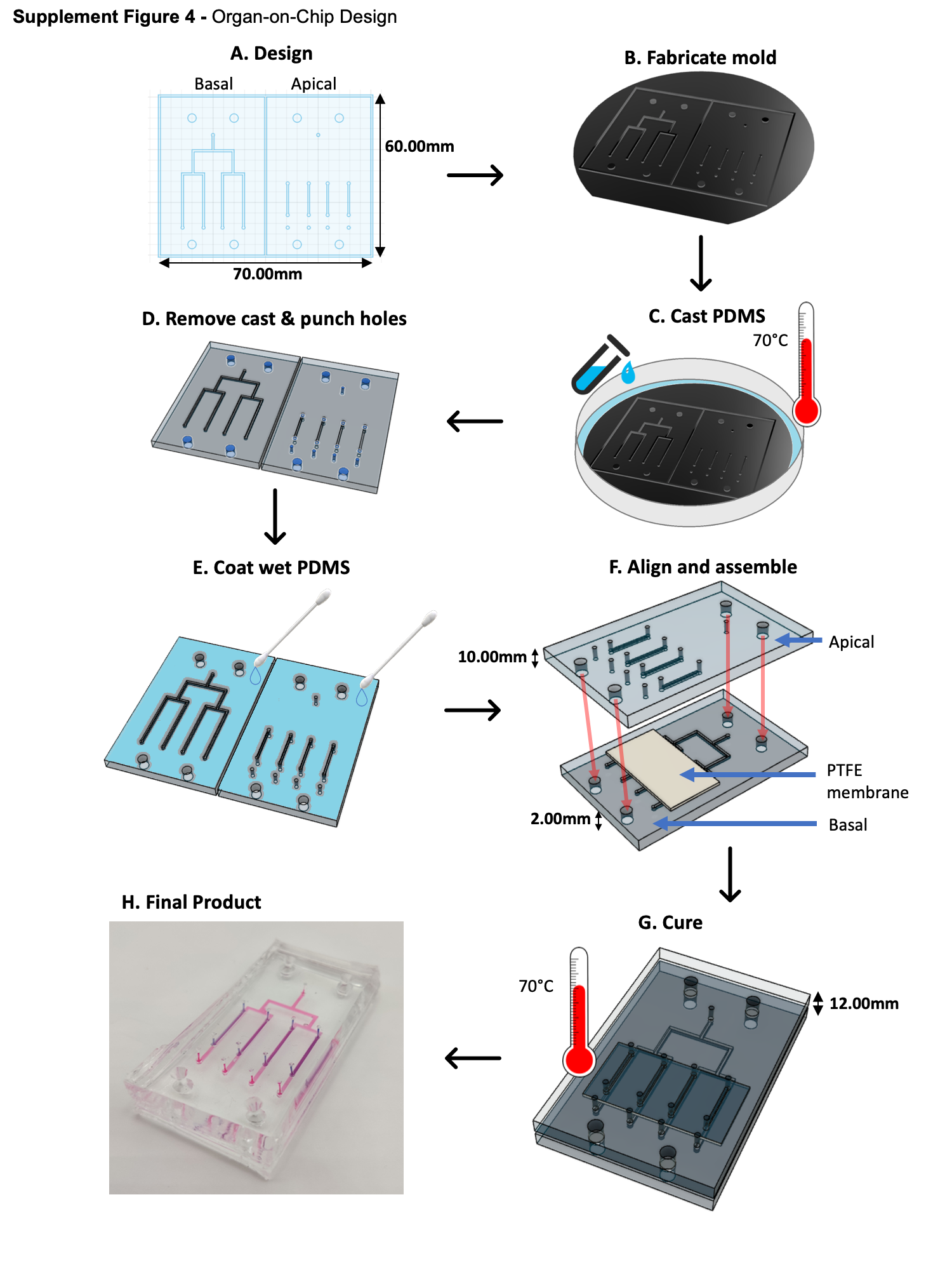

Supplement: Supplementary file 5 [file Image8.TIFF]

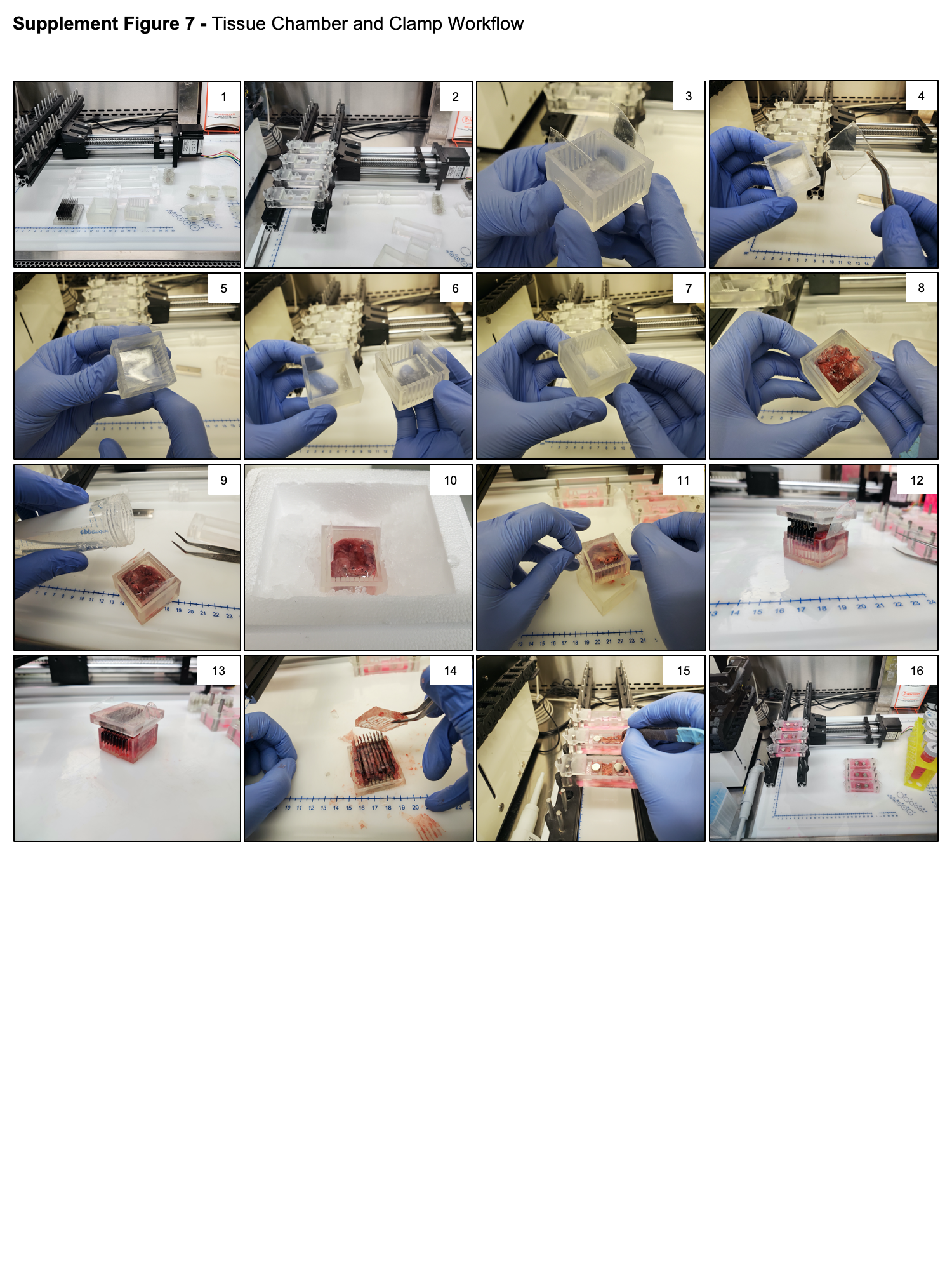

Supplement: Supplementary file 6 [file Image11.TIFF]

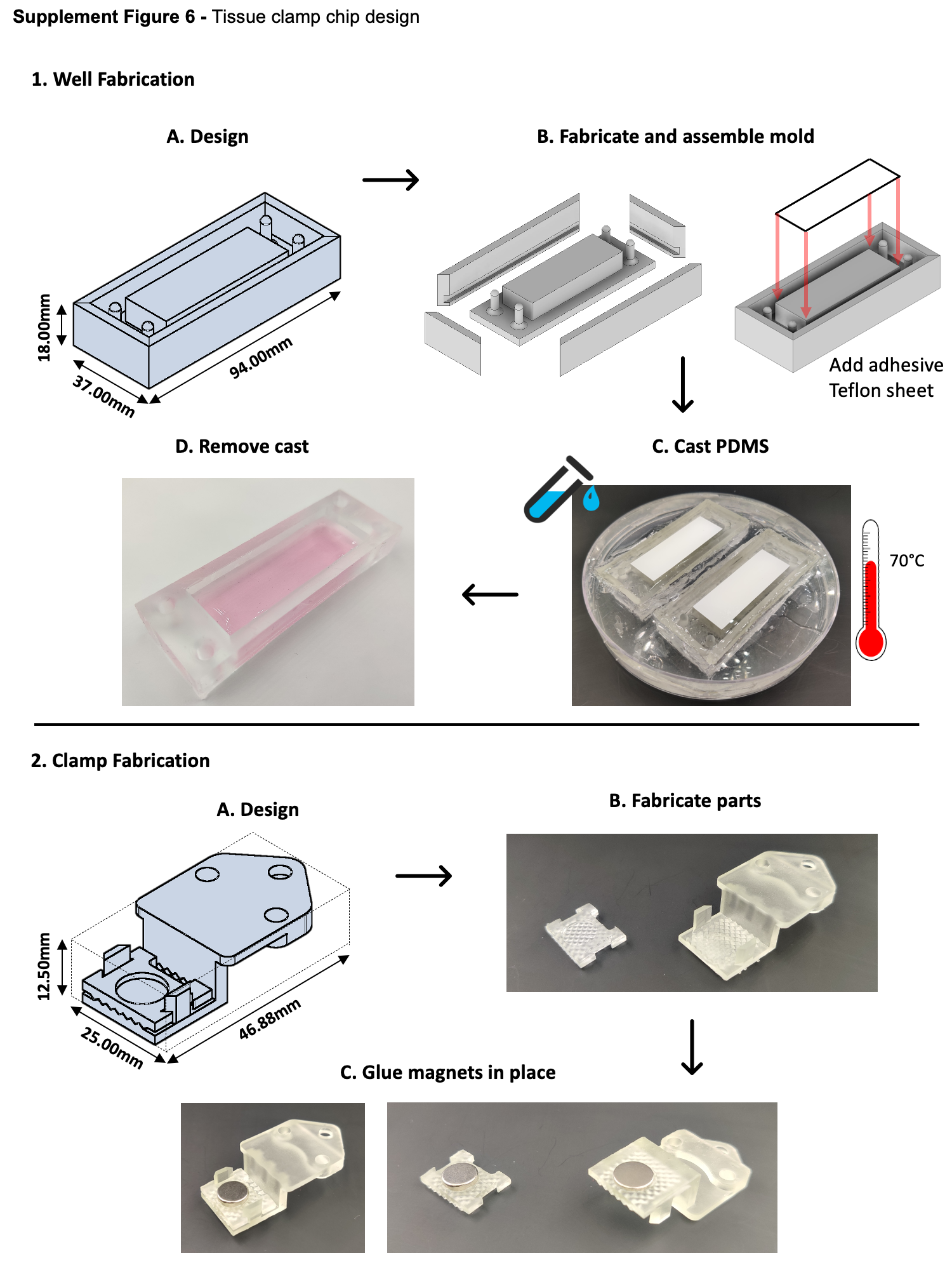

Supplement: Supplementary file 7 [file Image10.TIFF]

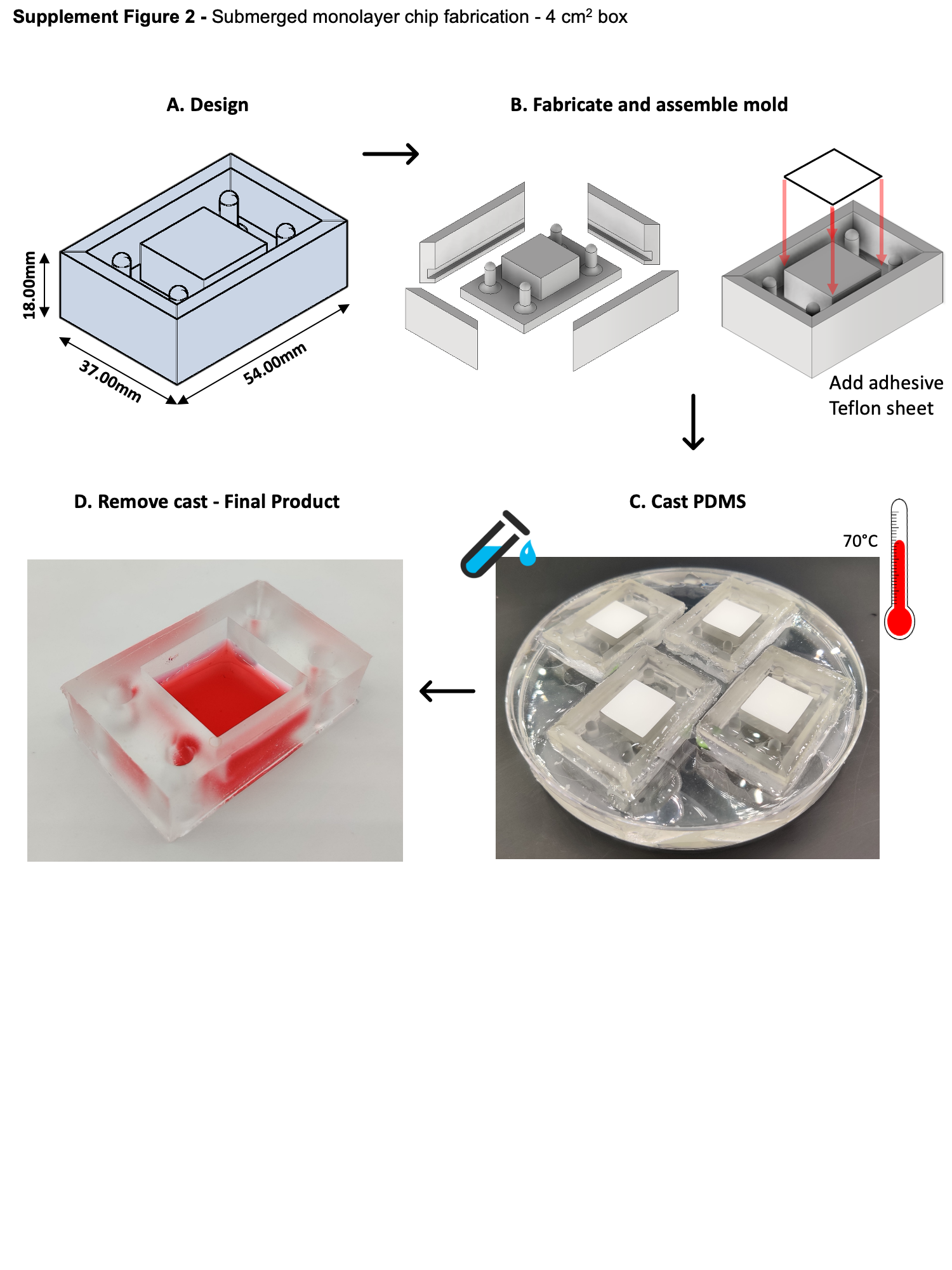

Supplement: Supplementary file 8 [file Image6.TIFF]

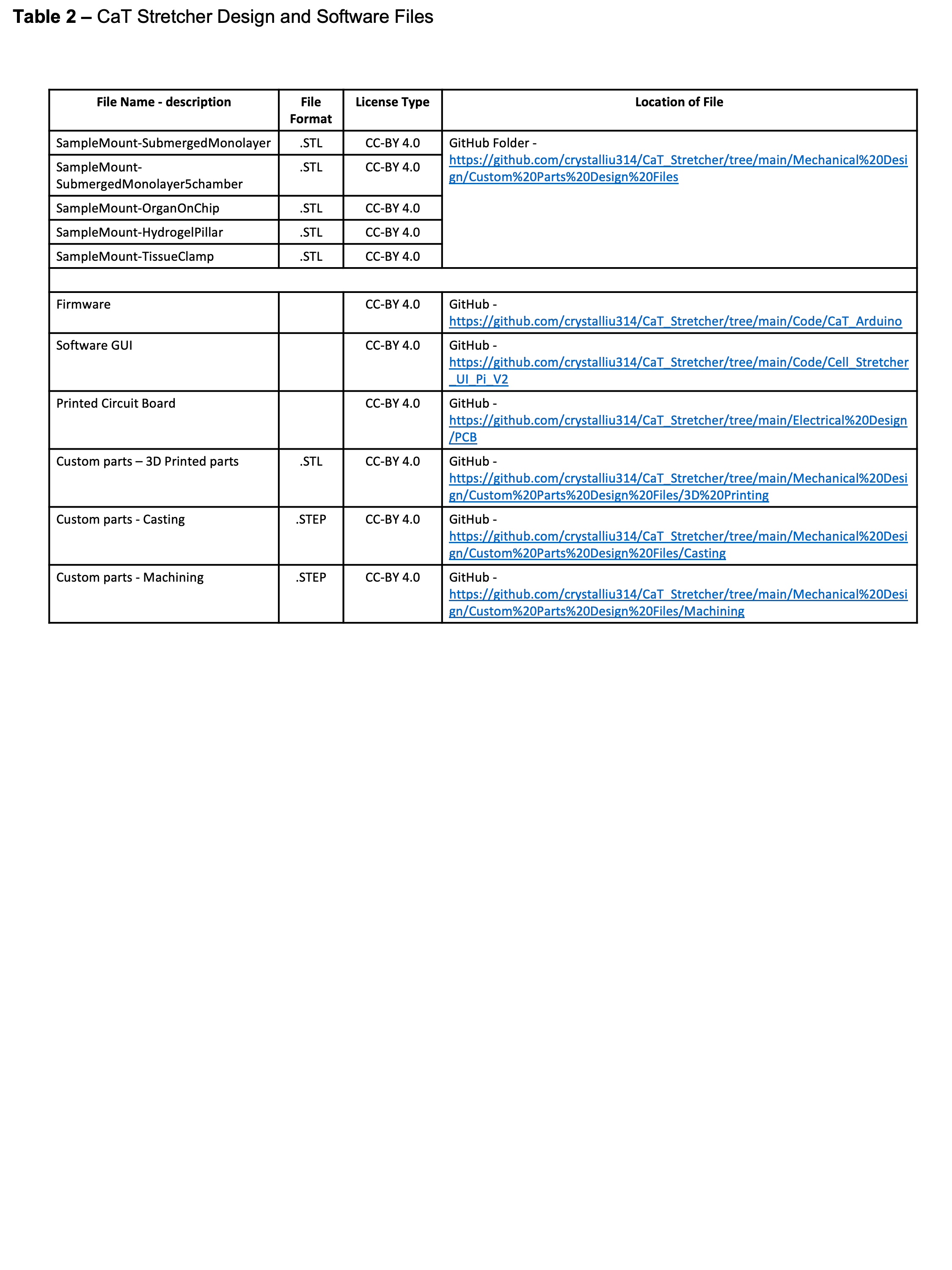

Supplement: Supplementary file 9 [file Image2.TIFF]

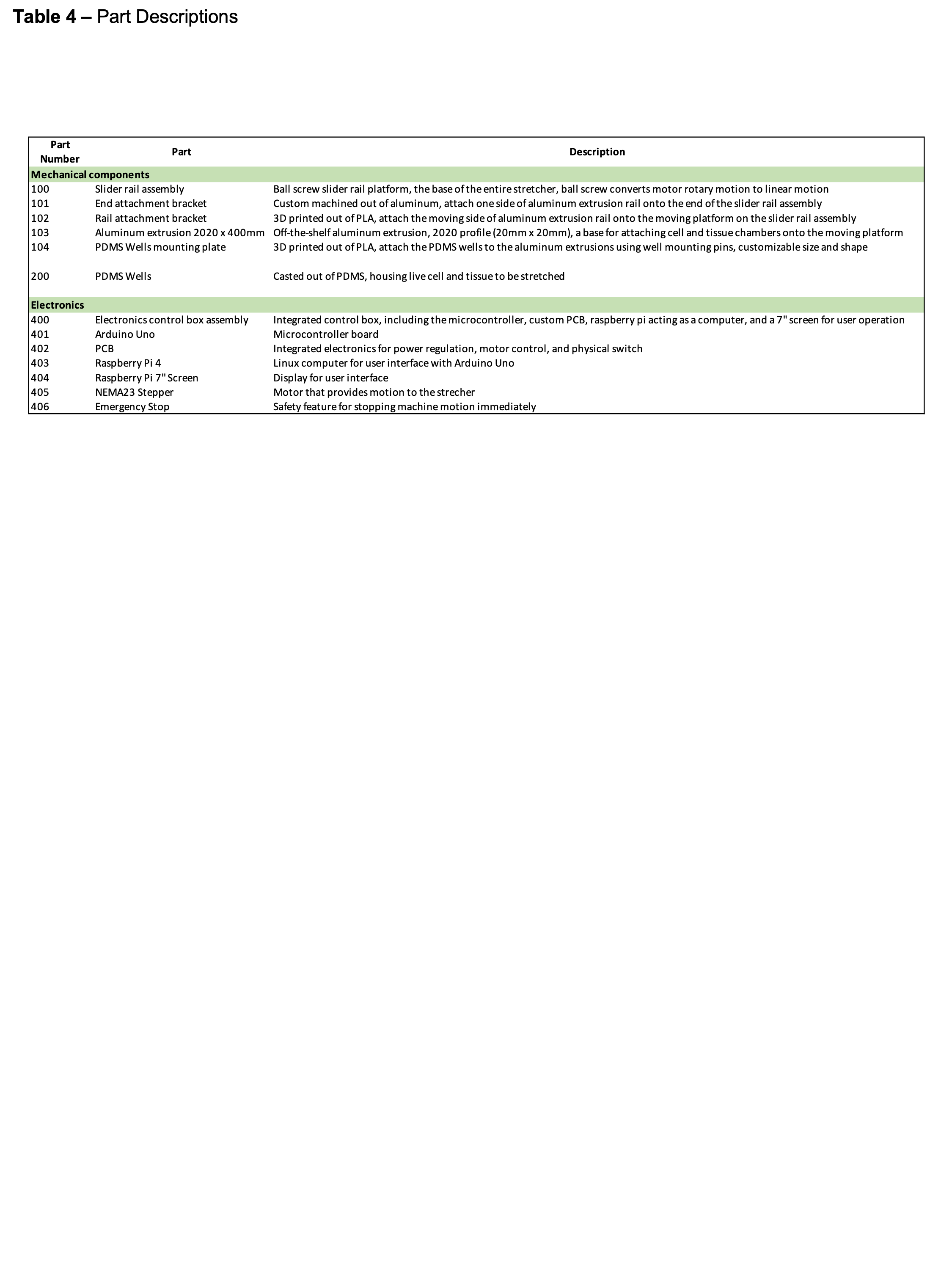

Supplement: Supplementary file 10 [file Image4.TIFF]

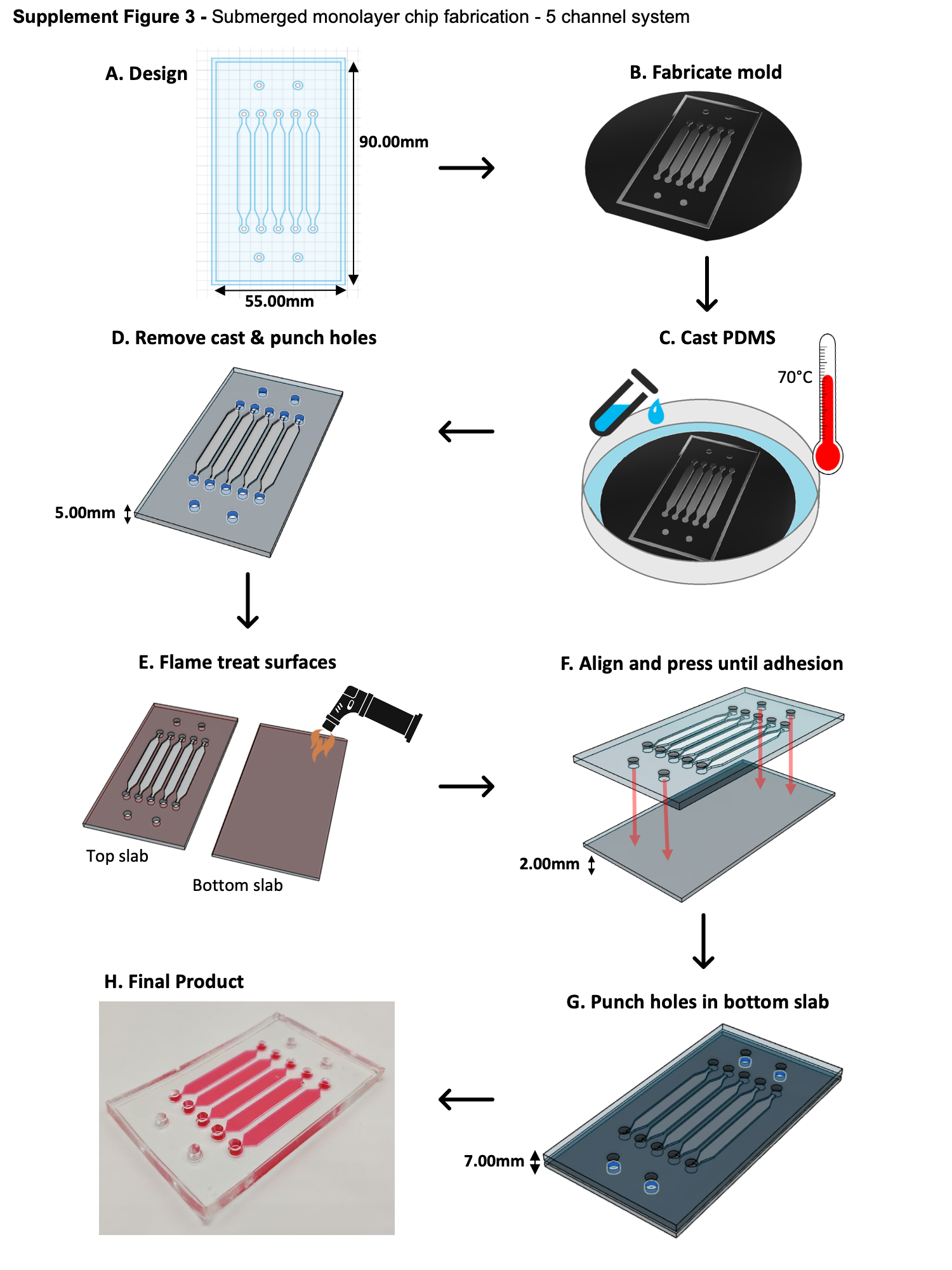

Supplement: Supplementary file 14 [file Image7.TIFF]
